# Comparing Kidney Health Outcomes in Children, Adolescents, and Adults With Focal Segmental Glomerulosclerosis

**DOI:** 10.1001/jamanetworkopen.2022.28701

**Published:** 2022-08-25

**Authors:** Debbie S. Gipson, Jonathan P. Troost, Cathie Spino, Samara Attalla, Joshua Tarnoff, Susan Massengill, Richard Lafayette, Virginia Vega-Warner, Sharon Adler, Patrick Gipson, Matthew Elliott, Frederick Kaskel, Damian Fermin, Marva Moxey-Mims, Richard N. Fine, Elizabeth J. Brown, Kimberly Reidy, Katherine Tuttle, Keisha Gibson, Kevin V. Lemley, Larry A. Greenbaum, Meredith A. Atkinson, Sangeeta Hingorani, Tarak Srivastava, Christine B. Sethna, Kevin Meyers, Cheryl Tran, Katherine M. Dell, Chia-shi Wang, Jennifer Lai Yee, Matthew G. Sampson, Rasheed Gbadegesin, J. J. Lin, Tammy Brady, Michelle Rheault, Howard Trachtman

**Affiliations:** 1 Division of Nephrology, Department of Pediatrics, University of Michigan, Ann Arbor; 2 Michigan Institute for Clinical and Health Research, University of Michigan, Ann Arbor; 3 School of Public Health, Department of Biostatistics, University of Michigan, Ann Arbor; 4 NephCure Kidney International, King of Prussia, Pennsylvania; 5 Division of Pediatric Nephrology, Department of Pediatrics, Levine Children’s Hospital, Atrium Health, Charlotte, North Carolina; 6 Department of Internal Medicine, Division of Nephrology, Stanford University, Palo Alto, California; 7 Division of Nephrology, Department of Internal Medicine, University of Michigan, Ann Arbor; 8 Division of Nephrology and Hypertension, Los Angeles Biomedical Research Institute at Harbor–University of California, Torrance; 9 Metrolina Nephrology Associates, Charlotte, North Carolina; 10 Division of Nephrology, Children’s Hospital at Montefiore; Albert Einstein College of Medicine, Bronx, New York; 11 Division of Nephrology, Children’s National Hospital, Department of Pediatrics, The George Washington University School of Medicine, Washington, DC; 12 Renaissance School of Medicine at Stony Brook University, Stony Brook University Medical Center, Stony Brook, New York; 13 Division of Nephrology, Department of Pediatrics, UT Southwestern Medical Center, Dallas, Texas; 14 Division of Nephrology, Department of Pediatrics, Albert Einstein College of Medicine, Montefiore Medical Center, New York, New York; 15 Providence Medical Research Center, Providence Health Care, Spokane, Washington; 16 Kidney Research Institute, Nephrology Division, and Institute for Translational Health Sciences, University of Washington, Seattle; 17 University of North Carolina Kidney Center at Chapel Hill; 18 Department of Pediatrics, USC Keck School of Medicine, Children’s Hospital Los Angeles, Los Angeles, California; 19 Division of Pediatric Nephrology, Department of Pediatrics, Emory University School of Medicine and Children’s Healthcare of Atlanta, Atlanta, Georgia; 20 Division of Pediatric Nephrology, Johns Hopkins University School of Medicine, Baltimore, Maryland; 21 Department of Pediatrics, University of Washington and Division of Nephrology, Seattle Children’s, Seattle; 22 Section of Nephrology, Children’s Mercy Hospital and University of Missouri at Kansas City; 23 Pediatric Nephrology, Cohen Children’s Medical Center of New York, Zucker School of Medicine at Hofstra/Northwell, Hempstead, New York; 24 Division of Nephrology, Children’s Hospital of Philadelphia, Philadelphia, Pennsylvania; 25 Children’s Center, Pediatric and Adolescent Medicine, Mayo Clinic, Rochester, Minnesota; 26 Center for Pediatric Nephrology, Cleveland Clinic Children’s, Cleveland, Ohio; 27 Division of Nephrology, Boston Children’s Hospital, Harvard Medical School, Boston, Massachusetts; 28 Kidney Disease Initiative, Broad Institute of MIT and Harvard, Cambridge, Massachusetts; 29 Pediatric Nephrology, Duke University Hospital, Durham, North Carolina; 30 Pediatric Nephrology, Wake Forest Baptist Health, Winston Salem, North Carolina; 31 Department of Pediatrics, Division of Nephrology, University of Minnesota, Minneapolis

## Abstract

**Importance:**

Focal segmental glomerulosclerosis (FSGS) is a common cause of end-stage kidney disease (ESKD) across the lifespan. While 10% to 15% of children and 3% of adults who develop ESKD have FSGS, it remains uncertain whether the natural history differs in pediatric vs adult patients, and this uncertainty contributes to the exclusion of children and adolescents in clinical trials.

**Objective:**

To examine whether there are differences in the kidney health outcomes among children, adolescents, and adults with FSGS.

**Design, Setting, and Participants:**

This cohort study used pooled and parallel analyses, completed July 5, 2022, from 3 complimentary data sources: (1) Nephrotic Syndrome Rare Disease Clinical Research Network (NEPTUNE); (2) FSGS clinical trial (FSGS-CT); and (3) Kidney Research Network (KRN). NEPTUNE is a multicenter US/Canada cohort study; FSGS-CT is a multicenter US/Canada clinical trial; and KRN is a multicenter US electronic health record–based registry from academic and community nephrology practices. NEPTUNE included 166 patients with incident FSGS enrolled at first kidney biopsy; FSGS-CT included 132 patients with steroid-resistant FSGS randomized to cyclosporine vs dexamethasone with mycophenolate; and KRN included 184 patients with prevalent FSGS. Data were collected from November 2004 to October 2019 and analyzed from October 2020 to July 2022.

**Exposures:**

Age: children (age <13 years) vs adolescents (13-17 years) vs adults (≥18 years). Covariates of interest included sex, disease duration, *APOL1* genotype, urine protein–to-creatinine ratio, estimated glomerular filtration rate (eGFR), edema, serum albumin, and immunosuppressive therapy.

**Main Outcomes and Measures:**

ESKD, composite outcome of ESKD or 40% decline in eGFR, and complete and/or partial remission of proteinuria.

**Results:**

The study included 127 (26%) children, 102 (21%) adolescents, and 253 (52%) adults, including 215 (45%) female participants and 138 (29%) who identified as Black, 98 (20%) who identified as Hispanic, and 275 (57%) who identified as White. Overall, the median time to ESKD was 11.9 years (IQR, 5.2-19.1 years). There was no difference in ESKD risk among children vs adults (hazard ratio [HR], 0.67; 95% CI, 0.43-1.03) or adolescents vs adults (HR, 0.85; 95% CI, 0.52-1.36). The median time to the composite end point was 5.7 years (IQR 1.6-15.2 years), with hazard ratio estimates for children vs adults of 1.12 (95% CI, 0.83-1.52) and adolescents vs adults of 1.06 (95% CI, 0.75-1.50).

**Conclusions and Relevance:**

In this study, the association of FSGS with kidney survival and functional outcomes was comparable at all ages.

## Key Points

**Question:**

Are kidney health outcomes different in children and adolescents compared with adults with focal segmental glomerulosclerosis (FSGS)?

**Findings:**

This cohort study with 482 patients from 3 independent cohorts found no significant differences by age in the median time to kidney failure, 11.9 years, or to the composite outcome of time to kidney failure or 40% reduction in kidney function, 5.7 years, despite differences in clinical context and medical management.

**Meaning:**

These findings suggest that the adverse outcomes of FSGS on kidney survival are severe and comparable across the lifespan.

## Teaser

This cohort study examines whether there are differences in the kidney health outcomes among children, adolescents, and adults with focal segmental glomerulosclerosis (FSGS).

## Introduction

Focal segmental glomerulosclerosis (FSGS) is one of the most common causes of end-stage kidney disease (ESKD), accounting for 10% to 15% of pediatric and 3% of adult ESKD population in the United States. There are no US Food and Drug Administration (FDA)–approved treatments for FSGS. While novel FSGS therapies are currently in various stages of clinical development, clinical trials usually focus on the adult patient population. Consequently, therapeutic development for affected children and adolescents with FSGS remains unaddressed.^1,2,3^ The exclusion of children and adolescents in drug development results in postmarketing, off-label drug use in minors without knowledge of age-appropriate dosing and safety information that is required for effective management.^4^

The inclusion of children and adolescents in clinical trials requires an understanding of the natural history and long-term outcomes of disease and safety considerations regarding the test therapy. This information can be used by regulatory agencies, such as the FDA and European Medicines Agency, to assess whether special study design and end point selection are needed for drug development plans that aim to include children and adolescents. Delineation of the short- and long-term impact of FSGS in pediatric vs adult patients is a key element in these deliberations. Estimates from the Toronto Glomerular Disease Registry suggest that the clinical course of FSGS is similar in children and adults.^5^ However, because of potential differences in disease presentation, evaluation, and utilization of therapy, the issue remains unresolved.

Therefore, we conducted this study to test the hypothesis that kidney survival and proteinuria outcomes in pediatric patients with FSGS are similar to their adult counterparts. We used 3 complementary data sets, including 1 prospective cohort study, 1 clinical trial, and 1 clinical data set.

## Methods

In this cohort study, analyzing a pooled data set from 3 existing data sources, the main outcomes of interest were ESKD, a composite of ESKD or 40% decline in estimated glomerular filtration rate (eGFR), and complete and/or partial remission of proteinuria. The primary exposure was age, categorized as child (<13 years), adolescent (13-17 years), and adult (≥18 years).

### Data Sources

Nephrotic Syndrome Rare Disease Clinical Research Network (NEPTUNE) is a prospective cohort study of primary proteinuric kidney diseases, including FSGS, that was launched in 2010. Patients were enrolled at the time of their first clinically indicated kidney biopsy.^6^ Data capture included demographic characteristics with patient-reported race and ethnicity, symptoms, coexisting conditions, hospitalizations, procedures, physical examination, medications, laboratory values, and patient-reported outcomes. In addition, urine, blood, and kidney biopsy tissue samples were stored in a biobank. The visit schedule included a baseline assessment within 30 days of the qualifying kidney biopsy and visits every 4 months during the first year and every 6 months thereafter for a maximum of 5 years. Race and ethnicity were collected as demographic variables to assess the representativeness of the studies with the affected population. Additionally, for National Institutes of Health (NIH)–sponsored studies, such as NEPTUNE, we are required to report demographic variables including race and ethnicity in our annual progress reports, thereby mandating these data collection items.

The FSGS clinical trial (FSGS-CT) was a multicenter randomized clinical trial of children and adults with steroid-resistant FSGS.^7^ The trial tested the efficacy on proteinuria of 52-week therapy with cyclosporine among 72 patients vs combination therapy with oral pulse dexamethasone and mycophenolate mofetil among 66 patients. Participants were enrolled in the US and Canada between November 2004 and May 2008. Race and ethnicity were self-reported. All patients received an angiotensin-converting enzyme inhibitor or angiotensin receptor blocker. Patients with no response by week 26 were declared nonresponders.

The Kidney Research Network (KRN) is an electronic health record (EHR)–based patient registry of children and adults with proteinuric kidney diseases in the United States, which was opened to enrollment in November 2015.^8,9^ Inclusion in this analysis was limited to patients with FSGS with a known date. KRN registry collects EHR data monthly, including demographic characteristics (with race and ethnicity extracted from the EHR), diagnoses, vital signs, laboratory results, medications, procedures, ESKD status, kidney biopsy reports, and vital status. Retrospective data were extracted from the earliest existing health record data point. The primary kidney diagnosis was confirmed by the patient’s nephrologist at the time of consent.

For NEPTUNE and FSGS-CT, the institutional review board (IRB) at each participating site approved the study protocol. For KRN, the protocol was reviewed and approved by the University of Michigan IRB, which serves as IRB of record for all enrolling sites. Informed consent and, as applicable, assent were obtained in accordance with IRB approval from each NEPTUNE (in-person), FSGS-CT (in-person), and KRN (in-person or via telephone) participant. This study followed Strengthening the Reporting of Observational Studies in Epidemiology (STROBE) reporting guideline.

### Clinical Methods

In NEPTUNE and KRN, laboratory tests were conducted locally except for serum creatinine and urinary protein excretion, which were measured centrally in NEPTUNE. In the FSGS-CT, all laboratory testing was performed centrally.

### Statistical Analysis

Baseline characteristics were compared by age category at time of first biopsy (KRN and NEPTUNE) or disease presentation (FSGS-CT) using medians and IQRs for continuous variables and frequencies and percentages for categorical variables. We subcategorized the pediatric population into 2 subgroups to reflect the common approach to regulatory considerations separating adolescents from younger children. This separation captures the potential impact of puberty on disease progression and clinical management compared with younger children. Characteristics studied include age; sex; race; ethnicity; Columbia FSGS pathology classification^10^; edema status; blood pressure (BP) and weight status categorized by latest guidelines^11,12,13^; urine protein–to-creatinine (UP:C) ratio; eGFR, calculated using the age- and creatinine-based U25 formula in those aged younger than 25 years and race-free 2021 Chronic Kidney Disease–Epidemiology Collaboration (CKD-EPI) creatinine equation for those aged 25 years or older^14,15,16,17^; serum albumin and lipid levels; prior immunosuppressive therapy (IST); and comorbidities. In addition, the following genetic and pathologic data from the NEPTUNE and FSGS-CT studies were included in this analysis: (1) *APOL1* genotype assessed directly via Sanger sequencing of the last 250 bases of exon 7; (2) evidence of monogenic disease based on a bioinformatics pipeline using stringent pathogenicity filtering^18,19^; and (3) the extent of interstitial fibrosis and global and segmental sclerosis on kidney biopsy. Participants with evidence of monogenic disease were excluded from primary analyses.

In the KRN cohort, laboratory data obtained within a month prior to the kidney biopsy were considered baseline. Comparisons of characteristics by age group were tested using Kruskal-Wallis tests for continuous variables and χ^2^ tests for categorical variables based on complete case analyses. No adjustments for multiplicity were made, as a penalty would bias the estimates toward the null and in favor of our hypotheses.

Practice patterns in the observational NEPTUNE and KRN studies were compared by age by reporting frequencies and percentages of treatments after biopsy. These analyses also tested for differences in likelihood of IST exposure after biopsy among those IST naive at biopsy using logistic regression. Unadjusted comparisons were made by age and after adjusting for UP:C ratio and eGFR.

Kaplan-Meier plots with log-ranked tests were used to compare the following end points by age: (1) time to ESKD, (2) time to ESKD or 40% reduction in eGFR, (3) time to complete remission (UP:C ratio <0.3 g/g), and (4) time to complete remission or partial remission defined as either a 40% reduction in UP:C ratio to a value less than 1.5 g/g or a 50% reduction to a value less than 3.5 g/g.^20^ Analyses were done for each of these end points except time to partial remission. In the KRN data set, proteinuria was commonly monitored based on screening dipstick testing and reflexive UP:C ratio per clinician preference. Given the substantial differences in proteinuria assessment in KRN compared with NEPTUNE and FSGS-CT, KRN was not included in the proteinuria analyses.

Tests for effect modification in time to ESKD or 40% reduction in eGFR and time to complete remission were performed using Cox proportional hazards models with interaction terms for subgroups of interest, as follows: sex, disease duration, *APOL1 *status, initial UP:C ratio, and treatment (in NEPTUNE and KRN, interactions by steroid and calcineurin inhibitor therapy were tested; in FSGS-CT, an interaction by randomized group was tested). Significant interactions would indicate whether the association between these characteristics and outcomes may differ by age.

Linear mixed-effects models with random slope (time) and intercept (participant) terms were used to compare trajectories of eGFR by age. Models used eGFR as the outcome, with an interaction term between time (years) and age category used to determine whether trajectories of eGFR differed by age. To handle hyperfiltration (defined as eGFR >120 mL/min/1.73 m^2^), a common finding among individuals with nephrotic syndrome, the analyses winsorized the eGFR distribution greater than 120 mL/min/1.73 m^2^ by imputing values greater than 120 mL/min/1.73 m^2^ to 120 mL/min/1.73 m^2^.

The analysis of the pooled sample represents the primary objective of the study, and the individual cohorts are exploratory. The comparisons between children vs adolescents vs adults and between children and adolescents vs adults were the primary focus. All other between-group comparisons that were performed for completeness were considered exploratory. Minimal detectable differences with 80% power overall and by cohort were assessed for each outcome (eTable 1 in the Supplement). To assess the validity of the model fit, we confirmed that the Cox models presented no violations of the proportional hazards assumption as assessed by the supremum test. Linear assumptions for continuous variables were assessed by martingale plots and showed no violations. Convergence criteria were met for all linear-mixed models. Analyses were conducted using SAS version 9.4 (SAS Institute). Two-tailed α = .05 was used to assess statistical significance

## Results

### Participant Characteristics

Flow diagrams for included NEPTUNE and KRN participants are shown in eFigures 1 and 2 in the Supplement. All randomized FSGS-CT participants were included except 6 with monogenic disease. Summary characteristics are reported in the Table, while eTables 2, 3, and 4 in the Supplement report sample-specific details. NEPTUNE included 166 participants (32 children, 29 adolescents, and 105 adults); FSGS-CT included 132 (42 children, 48 adolescents, and 42 adults); KRN included 184 (53 children, 25 adolescents, and 106 adults). Overall, the study included 127 (26%) children, 102 (21%) adolescents, and 253 (52%) adults, including 215 (45%) female participants and 138 (29%) who identified as Black, 98 (20%) who identified as Hispanic, and 275 (57%) who identified as White.

**Table.** Sample Characteristics

Characteristic	Participants, No. (%)			
	NEPTUNE	FSGS-CT	KRN	Pooled
Study design	Prospective cohort	Randomized clinical trial	Electronic medical record–based registry	NA
Inclusion criteria	Incident FSGS enrolled at first kidney biopsy	Steroid-resistant FSGS	Prevalent FSGS patients	NA
Location	United States and Canada	United States and Canada	United States	NA
Enrollment	2010-2019^a^	2004-2008	2015-2019^a^	NA
Sample size, No.	166	132	184	482
Age, median (IQR), y	30 (14-50)	15 (11-23)	22 (9-42)	19 (12-38)
Children (age <13 y)	32 (19)	42 (32)	53 (29)	127 (26)
Adolescents (13-17 y)	29 (17)	48 (36)	25 (14)	102 (21)
Adults (≥18 y)	105 (64)	42 (32)	106 (58)	253 (52)
Sex				
Female	67 (40)	61 (46)	87 (47)	215 (45)
Male	99 (60)	71 (54)	97 (53)	258 (55)
Race and ethnicity				
Asian	10 (6)	3 (2)	16 (9)	29 (6)
Black	60 (37)	50 (38)	28 (15)	138 (29)
Native American or Pacific Islander	0	2 (1)	0	2 (<0.1)
White	85 (54)	75 (57)	115 (63)	275 (57)
Other	4 (2)	2 (1)	21 (11)	27 (6)
Unknown, No.	7	0	0	7
Hispanic ethnicity	36 (22)	23 (17)	39 (21)	98 (20)
*APOL1* genotype				
2 Risk alleles	30 (20)	25 (23)	NA	NA
0-1 Risk alleles	117 (80)	86 (77)	NA	NA
Unknown, No.	19	21	184	224
Interstitial fibrosis, median (IQR), %	15 (3-27)	10 (4-25)	NA	NA
Global sclerosis, median (IQR), %	10 (0-39)	0 (0-12)	NA	NA
Segmental sclerosis, median (IQR), %	7 (3-16)	20 (9-33)	NA	NA
Baseline UP:C ratio, median (IQR), g/g	3.8 (1.9-7.5)	4.0 (2.2-8.4)	4.0 (1.7-7.6)	3.9 (1.9-7.6)
Baseline eGFR, median (IQR), mL/min/1.73 m^2^	72 (46-95)	82 (60-116)	64 (33-105)	80 (54-105)
ESKD or 40% reduction in eGFR by year 3, %				
Children	27	40	30	31
Adolescents	31	38	38	36
Adults	38	40	28	33
ESKD by year 3, %				
Children	0	13	13	11
Adolescents	0	22	21	16
Adults	12	23	24	20
Complete remission by month 6, %				
Children	22	32	NA	27
Adolescents	28	20	NA	22
Adults	16	10	NA	14
Complete or partial remission by month 6, %				
Children	55	56	NA	56
Adolescents	42	57	NA	52
Adults	44	62	NA	50

Abbreviations: eGFR, estimated glomerular filtration rate; FSGS, focal segmental glomerulosclerosis; FSGS-CT, focal segmental glomerulosclerosis clinical trial; KRN, Kidney Research Network; NA, not applicable; NEPTUNE, Nephrotic Syndrome Study Network; UP:C, urine protein–to-creatinine.

^a^ Ongoing. Censored at 2019 for these analyses.

Baseline eGFR was highest among children, lowest among adults, with adolescents in between. However, more children had nephrotic syndrome compared with adolescents and adults at baseline. Children had the lowest extent of interstitial fibrosis, followed by adolescents and then adults. Global sclerosis was lowest among children. Overall, 55 (21%) had 2 *APOL1* risk alleles. Administration of agents to block the renin–angiotensin–aldosterone system was more prevalent in adult patients (eTables 2-4 in the Supplement). In contrast, children were more likely to have received IST prior to biopsy in NEPTUNE and KRN. Pretrial IST was required and limited to corticosteroids in FSGS-CT (eTable 5 in the Supplement). In analyses confined to NEPTUNE, where nearly all participants had proteinuria and eGFR data available at time of biopsy, there was no difference in IST by age in unadjusted analyses (eTable 6 in the Supplement).

### Progression to Kidney Failure

**Figure 1.**
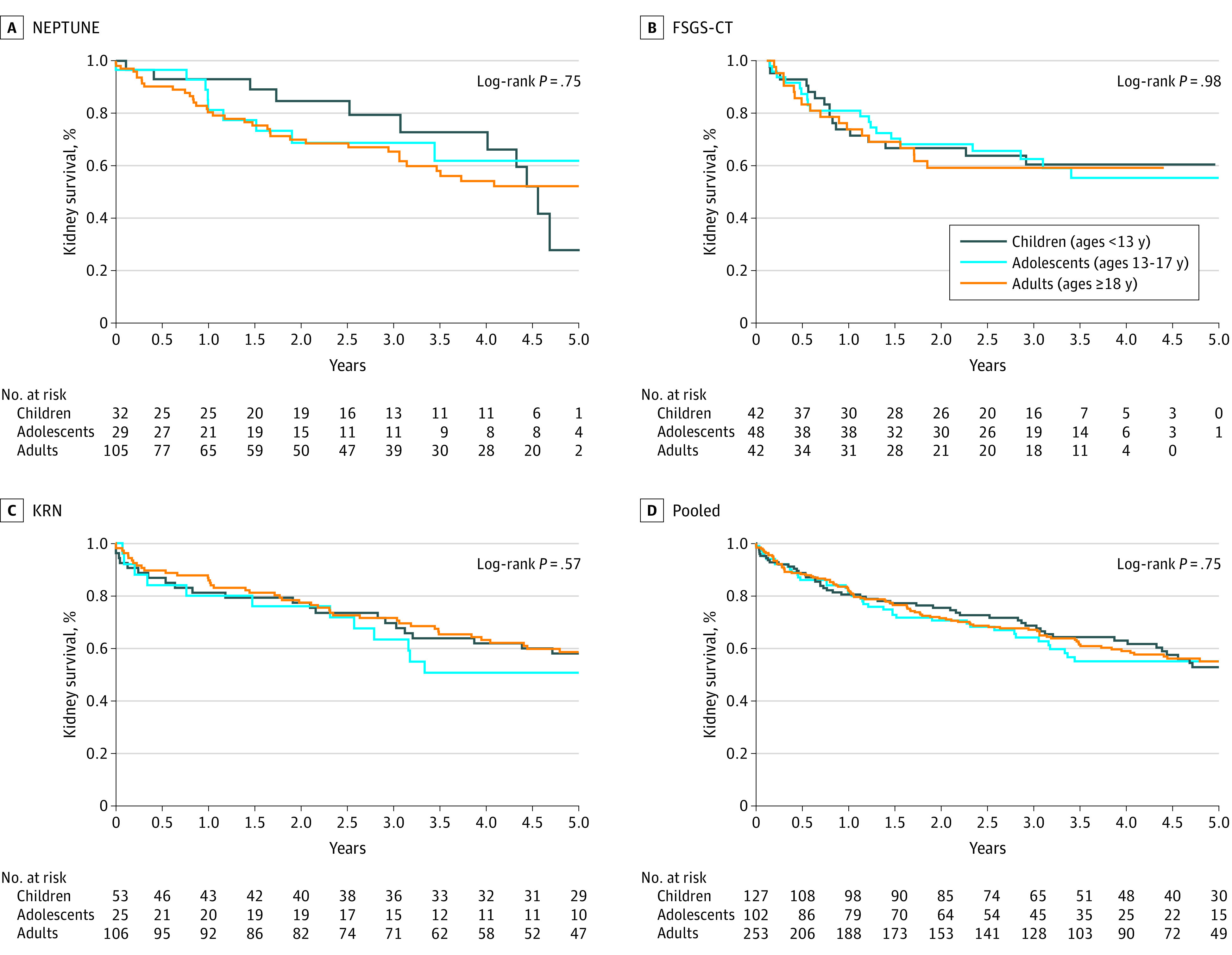
Time to End-Stage Kidney Disease or 40% Reduction in Estimated Glomerular Filtration Rate by Age Nephrotic Syndrome Rare Disease Clinical Research Network (NEPTUNE) included 166 participants, with 56 events; Focal segmental glomerulosclerosis clinical trial (FSGS-CT) included 132 participants, with 52 events; Kidney Research Network (KRN) included 184 participants, with 121 events. The pooled analysis included 482 participants with 229 events.

Figure 1 contains Kaplan-Meier curves of time to the composite ESKD or 40% reduction in eGFR. The pooled median time to progression was 5.7 years (IQR, 1.6-15.2 years). There was no difference in progression by age group (children vs adults: HR, 1.12; 95% CI, 0.83-1.52; adolescents vs adults: HR, 1.06; 95% CI, 0.75-1.50). Time to ESKD is shown in eFigures 3 and 4 in the Supplement. (eFigure 4 in the Supplement shows KRN estimates extended to 15 years.) Pooled median time to ESKD was 11.9 years (IQR, 5.2-19.1 years). Pooled analysis showed no difference in time to ESKD by age group (children vs adults: hazard ratio [HR], 0.67; 95% CI, 0.43-1.03; adolescents vs adults: HR, 0.85; 95% CI, 0.52-1.36) (eFigure 3D in the Supplement). In NEPTUNE, no children or adolescents progressed to ESKD within 5 years (eFigure 3A in the Supplement). There was no difference in progression to kidney failure by age in FSGS-CT at 5 years or in KRN at 5 or 15 years (eFigures 3B, 3C, and 4 in the Supplement).

Sensitivity analyses including monogenic patients in the models are shown in eTable 7 in the Supplement. There were no differences by age after covariate adjustment, and no significant change in results after including monogenic patients.

Tests of effect modification by sex, disease duration, *APOL1* risk genotype, initial UP:C ratio, and treatment are shown in eTable 8 and eFigure 5 in the Supplement. There was subgroup variation in time to the composite outcome of ESKD or 40% eGFR reduction in the NEPTUNE sample by nephrotic vs nonnephrotic proteinuria at baseline. Nephrotic-range children appeared to have less progression. Among those with subnephrotic proteinuria, progression was similar by age (eFigure 6 in the Supplement).

### Kidney Function Over Time

**Figure 2.**
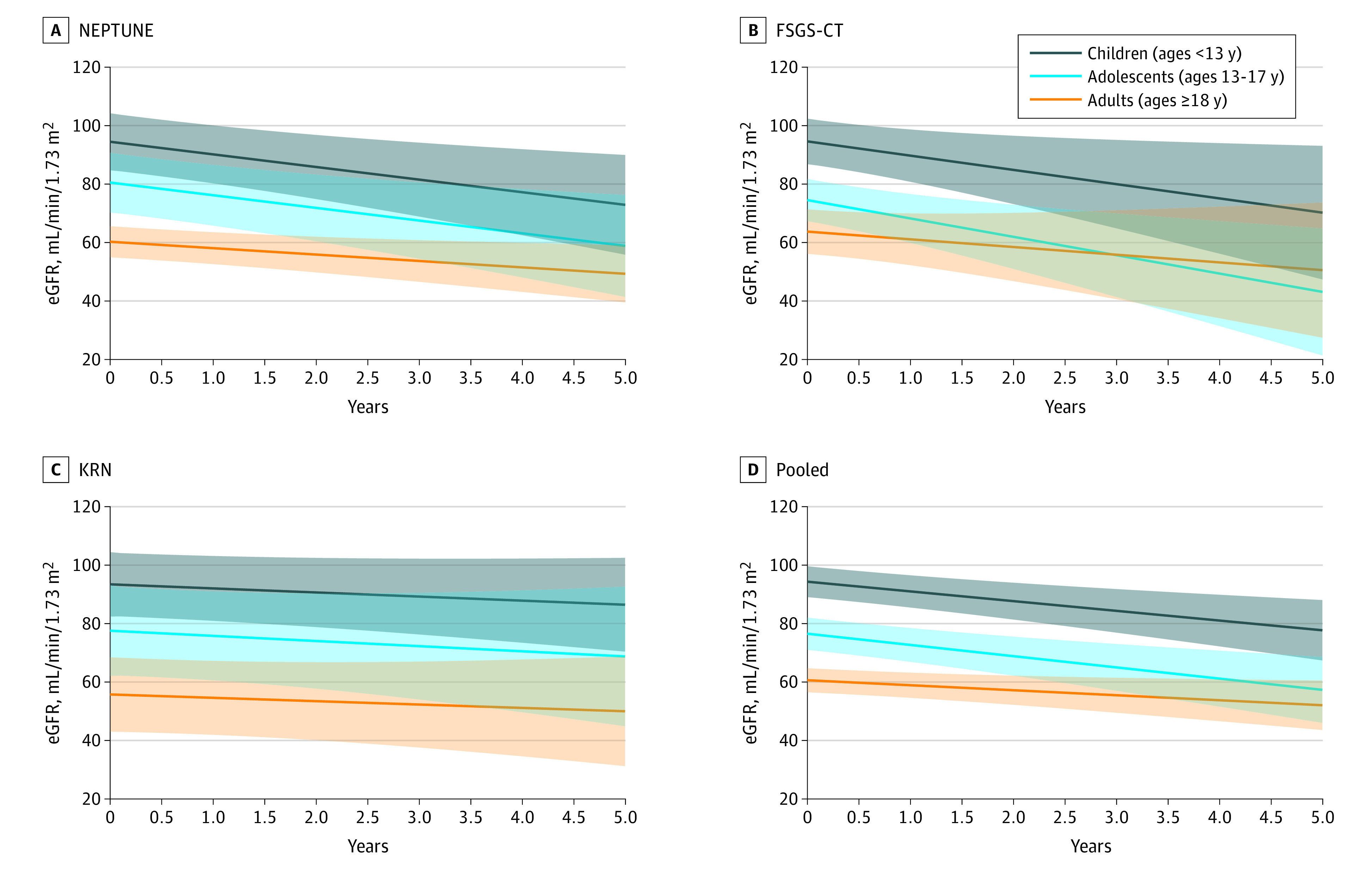
Results of Linear Mixed-Effects Models of Estimated Glomerular Filtration Rate (eGFR) Values shown are regression estimates, with shaded areas indicating 95% CIs. Nephrotic Syndrome Rare Disease Clinical Research Network (NEPTUNE) included 166 participants, with 1827 observations; focal segmental glomerulosclerosis clinical trial (FSGS-CT) included 132 participants, with 1992 observations; Kidney Research Network (KRN) included 184 participants, with 3082 observations. The pooled analysis included 482 participants, with 6901 observations.

Average eGFR trajectories derived from mixed models are shown in Figure 2, and model estimates are found in eTable 9 in the Supplement. In pooled analysis, adults had an estimated slope of −1.71 mL/y (95% CI, −3.23 to −0.19 mL/y); adolescents, −3.84 mL/y (95% CI, −5.86 to −1.82 mL/y); children, −3.32 mL/y (95% CI, −5.13 to −1.51 mL/y). Children and adolescents had higher starting eGFR than adults, but there was no statistically significant difference in the rate of decline in eGFR over time in the pooled analysis.

### Proteinuric Remission

**Figure 3.**
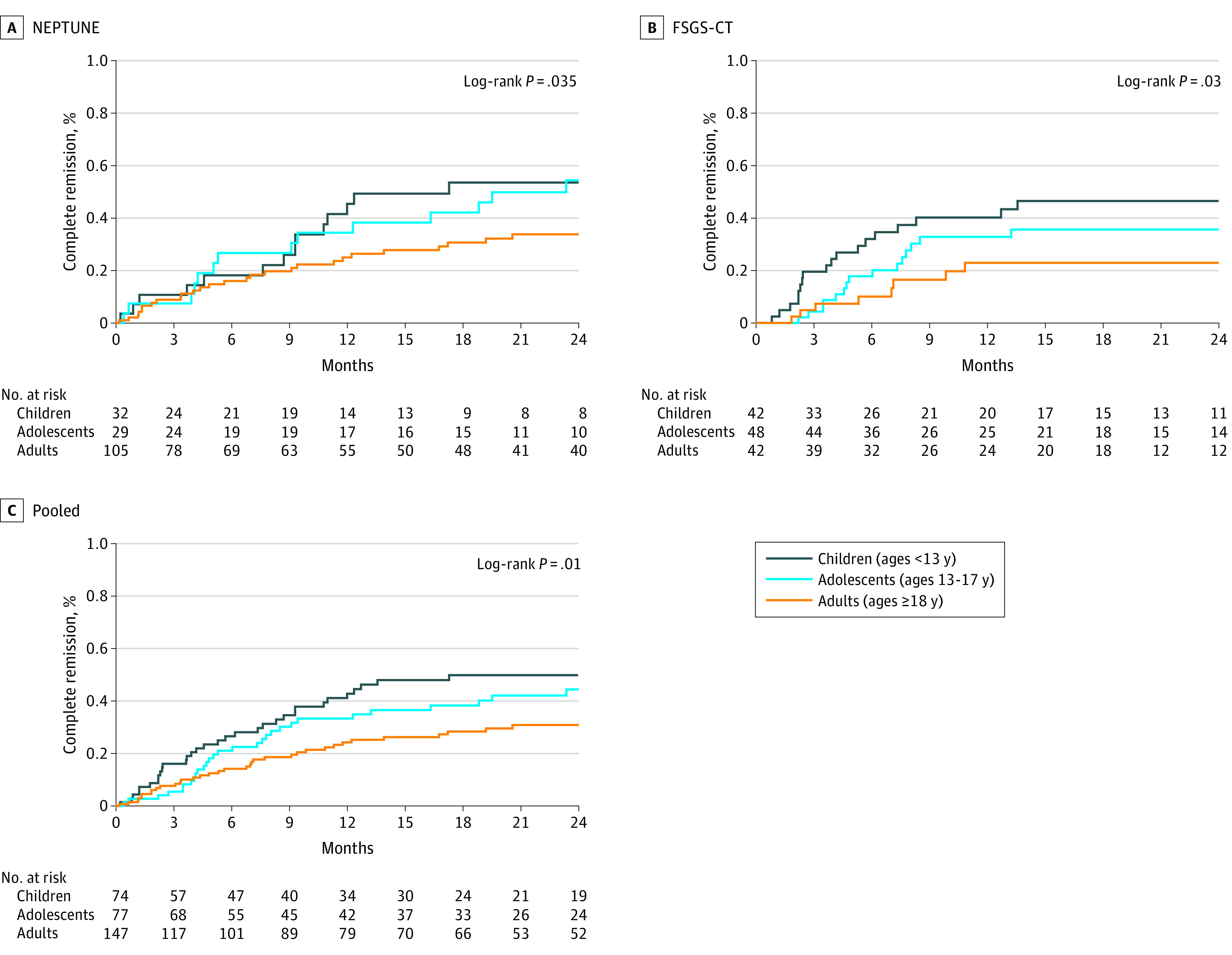
Time to Complete Remission by Age Nephrotic Syndrome Rare Disease Clinical Research Network (NEPTUNE) included 166 participants, with 64 events; focal segmental glomerulosclerosis clinical trial (FSGS-CT) included 132 participants, with 50 events. The pooled analysis included 298 participants with 114 events.

**Figure 4.**
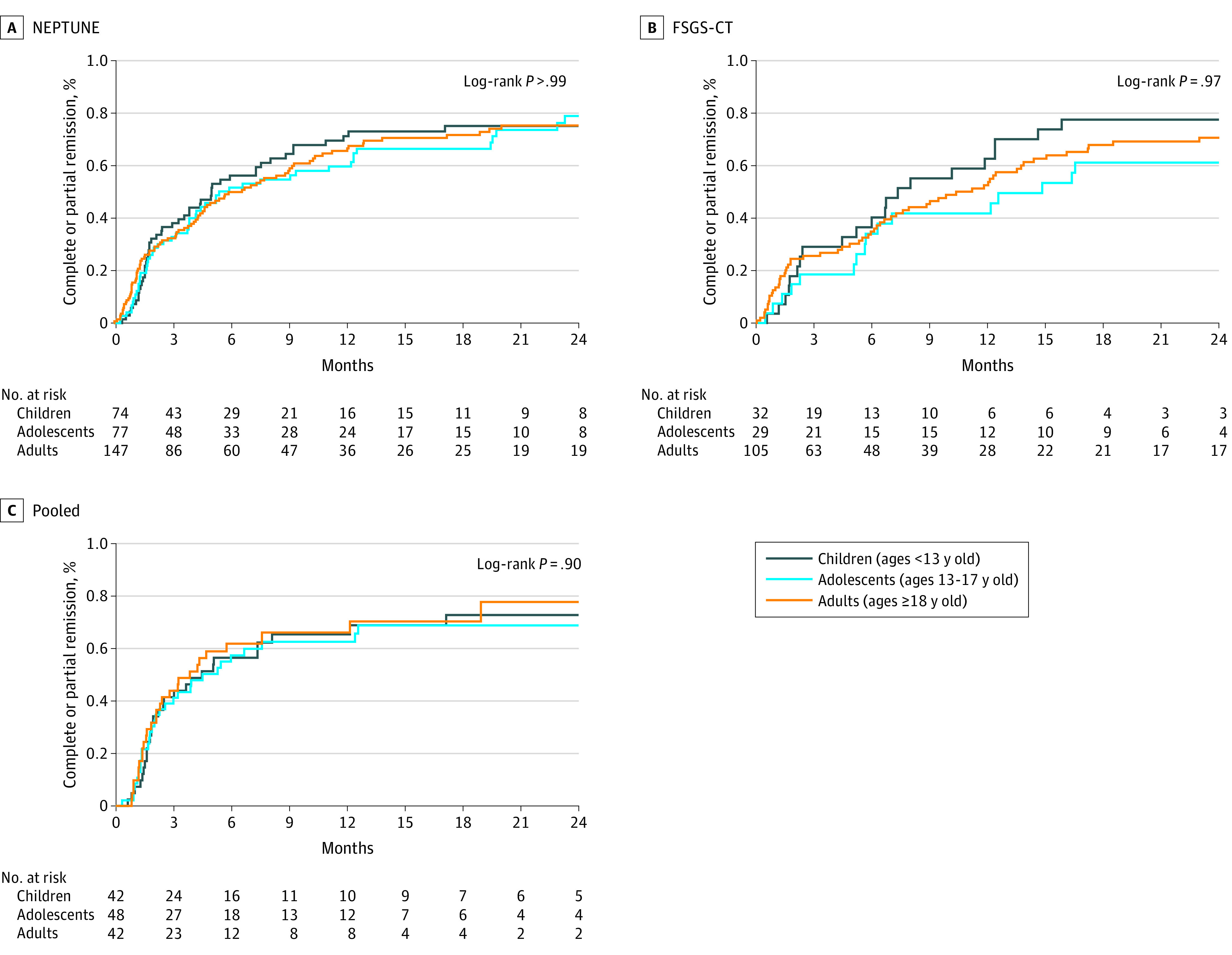
Time to Complete Remission or Urine Protein–to-Creatine Ratio Less Than 1.5 g/g and 40% Reduction in Urine Protein–to-Creatine Ratio by Age Nephrotic Syndrome Rare Disease Clinical Research Network (NEPTUNE) included 166 participants, with 114 events; focal segmental glomerulosclerosis clinical trial (FSGS-CT) included 132 participants, with 88 events. The pooled analysis included 298 participants with 202 events.

In pooled analysis of NEPTUNE and FSGS-CT only, adults were less likely to achieve complete remission than adolescents or children (Figure 3). By 12 months, 49% (95% CI, 30%-69%) of children, 38% (95% CI, 20%-57%) of adolescents, and 25% (95% CI, 16%-34%) of adults had reached complete remission. There was no difference in time to the composite complete or novel FSGS partial remission end point (Figure 4) or to a conventional partial remission end point, namely 50% reduction in UP:C ratio to a level of less than 3.5 g/g (eFigure 7 in the Supplement) by age. There was no evidence of effect modification by clinical characteristics and treatment type (eTable 8 in the Supplement).

## Discussion

This report describes the natural history of patients with FSGS in a pooled analysis of 482 participants with FSGS from 3 contemporary cohorts. Although there were differences in clinical presentation and treatment patterns in child, adolescent, and adult patients, the disease course based on the composite kidney health outcome of a 40% reduction in eGFR or ESKD was remarkably similar in the 3 groups. To our knowledge, this report is unique in that it captures patients from across North America and represents populations from 3 distinct settings: a cohort study, a randomized clinical trial, and clinical data from medical practices across the United States. As such, it reduces the magnitude of any unique design-related bias that may have influenced previous publications that focused on a single population. The ability to compare kidney function outcomes in pediatric vs adult patients with FSGS in such diverse contexts represents a unique feature of our study that enhances its external validity.

Children tended to present with overt nephrotic syndrome, less interstitial fibrosis in the kidney, and higher eGFR than adolescents or adults. In addition, they were more likely to receive IST. Nevertheless, all age groups lost kidney function at a comparable rate over time, and the majority of patients in each age group did not achieve a complete proteinuria remission. The 5-year kidney survival in pediatric patients with FSGS in the pooled analysis was comparable with published outcomes from the pediatric steroid-resistant nephrotic syndrome PodoNET registry.^21^

The biological context in which FSGS develops is undoubtedly different within the full spectrum of affected patients. Individuals with a monogenic etiology of FSGS are usually younger and less likely to respond to treatment.^21^ After excluding patients with known monogenic disease, the natural history of FSGS was comparable in pediatric vs adult patients. Adult patients may have a higher burden of comorbid conditions that theoretically may accelerate the progression of kidney disease. However, our findings suggest that despite these potential differences, in the aggregate, the clinical course of FSGS is similar across the lifespan.

It is increasingly acknowledged that the diagnosis of FSGS, which is based on histopathological findings, provides limited information about prognosis and appropriate therapy. FSGS is recognized to be a heterogeneous group of disorders with distinct molecular injury pathway(s).^22^ These pathogenetic subtypes are likely to occur in all ages, contribute to the poor outcomes of FSGS, and provide justification for drug development of targeted therapies in mechanistically defined patients regardless of age.^23^

There is a well-documented shortfall in the performance of adequately designed and powered clinical trials in nephrology. This is especially the case for pediatric patients. A review of ClinicalTrials.gov (accessed June 24, 2022) identified 12 ongoing trials for FSGS. Of these, 5 of 12 (42%) are open to the enrollment of children and adolescents, 3 of which are testing a single agent. A key first step in expanding drug development for all ages is an accurate delineation of natural history and relevant health outcomes for the disease being studied. Our finding that key kidney function outcomes in patients with FSGS are poor and comparable in children, adolescents, and adults highlights the unmet clinical need across the lifespan. This information clearly needs to be supplemented by information about the safety of the therapeutic agent under investigation that is germane to pediatric patients. Nevertheless, this study provides essential information to inform clinical trial design for specific and combined age groups.

### Strengths and Limitations

FSGS is a rare glomerular disease and our overall cohort is one of the largest and diverse with comprehensive assessment of long-term kidney function. However, there are limitations to this study. First, the sample size in pooled analysis was modest and may have limited the power to detect differences in outcomes. For example, we were only powered for an HR difference of 0.56 in the pooled analyses of time to ESKD comparing children vs adults. In fact, we observed an HR of 0.67, a difference in magnitude that would be clinically significant, if confirmed with larger sample size, and suggest that children have slower progression to ESKD despite having no difference in eGFR slope or time to the composite end point of 40% eGFR loss or ESKD. However, we posit that this discrepancy is instead because children had higher initial levels of eGFR. Most of the remaining HR results were close to unity. Second, we cannot be certain that the underlying pathogenetic mechanisms of FSGS are similarly distributed across the age groups. However, the cohorts reflect contemporary approaches to the diagnosis, classification, and management of FSGS in children and adults. Third, there was incomplete harmonization of the data collection across the data sources. Genetic testing was not performed in all patients. Consequently, the exclusion of patients with monogenic FSGS was likely incomplete. Sensitivity analysis obtained similar results with inclusion or exclusion of patients with known monogenic causes of FSGS.

## Conclusions

This report found a reasonably consistent clinical trajectory under current standard of care therapies for FSGS. We have quantified the uncertainty in the estimates to inform trial design and support decision-making. Our findings provide evidence of kidney disease progression trajectories for children, adolescents, and adults with FSGS that can be used to strengthen clinical trial design and extrapolation studies. This should facilitate selection of population, end points, and analysis plan when designing clinical trials of potential novel therapies for children, adolescents, and adults with FSGS.

## Notes

Supplement. **eTable 1.** Minimal Detectable Differences by Outcome With 80% Power and 2-Sided Alpha **eTable 2.** NEPTUNE: Baseline Demographic, Clinical, Genetic, and Laboratory Characteristics of Participants With FSGS by Age **eTable 3.** FSGS-CT: Baseline Demographic, Clinical, Genetic, and Laboratory Characteristics of Randomized Participants by Age **eTable 4.** KRN: Baseline Demographic, Clinical, and Laboratory Characteristics of Participants by Age at FSGS Biopsy **eTable 5. **Pooled (NEPTUNE, KRN): Medication Exposures Before and After Biopsy by Age **eTable 6.** NEPTUNE: Logistic Regression Models of Immunosuppressive Therapy Treatment (Treated vs Untreated) Among Participants Who Were Treatment Naive At Biopsy **eTable 7.** Sensitivity Analyses of Potential Confounding and Excluding Monogenic Disease **eTable 8.** Tests of Effect Modification in Cox Proportional Hazard Models of Time to ESKD or 40% Reduction in eGFR and of Time to Complete Remission by Study **eTable 9.** Results of Linear-Mixed Effects Models of eGFR After Biopsy by Study and Pooled **eFigure 1.** NEPTUNE: Participants Included in NEPTUNE Analyses **eFigure 2.** KRN: Participants Included in KRN Analyses **eFigure 3.** Time to ESKD by Age **eFigure 4.** KRN: Time to ESKD by Age **eFigure 5.** Time to ESKD or 40% Reduction in eGFR by Age: Significant Effect Modification by Sex in FSGS-CT **eFigure 6.** Time to ESKD or 40% Reduction in eGFR by Age: Significant Effect Modification by Baseline UP:C Ratio in NEPTUNE **eFigure 7.** Time To Complete or Partial Remission by Age (Partial Remission Defined as UP:C Ratio <3.5 g/g and 50% Reduction in UP:C Ratio From Baseline)
