# Supplementary material for: Comparing Kidney Health Outcomes in Children, Adolescents, and Adults With Focal Segmental Glomerulosclerosis
Source: JAMA Netw Open. 2022 Aug 25;5(8):e2228701. doi: 10.1001/jamanetworkopen.2022.28701 (PMC9412226; doi:10.1001/jamanetworkopen.2022.28701)
Supplement: Supplement. — eTable 1. Minimal Detectable Differences by Outcome With 80% Power and 2-Sided Alpha eTable 2. NEPTUNE: Baseline Demographic, Clinical, Genetic, and Laboratory Characteristics of Participants With FSGS by Age eTable 3. FSGS-CT: Baseline Demographic, Clinical, Genetic, and Laboratory Characteristics of Randomized Participants by Age eTable 4. KRN: Baseline Demographic, Clinical, and Laboratory Characteristics of Participants by Age at FSGS Biopsy eTable 5. Pooled (NEPTUNE, KRN): Medication Exposures Before and After Biopsy by Age eTable 6. NEPTUNE: Logistic Regression Models of Immunosuppressive Therapy Treatment (Treated vs Untreated) Among Participants Who Were Treatment Naive At Biopsy eTable 7. Sensitivity Analyses of Potential Confounding and Excluding Monogenic Disease eTable 8. Tests of Effect Modification in Cox Proportional Hazard Models of Time to ESKD or 40% Reduction in eGFR and of Time to Complete Remission by Study eTable 9. Results of Linear-Mixed Effects Models of eGFR After Biopsy by Study and Pooled eFigure 1. NEPTUNE: Participants Included in NEPTUNE Analyses eFigure 2. KRN: Participants Included in KRN Analyses eFigure 3. Time to ESKD by Age eFigure 4. KRN: Time to ESKD by Age eFigure 5. Time to ESKD or 40% Reduction in eGFR by Age: Significant Effect Modification by Sex in FSGS-CT eFigure 6. Time to ESKD or 40% Reduction in eGFR by Age: Significant Effect Modification by Baseline UP:C Ratio in NEPTUNE eFigure 7. Time To Complete or Partial Remission by Age (Partial Remission Defined as UP:C Ratio <3.5 g/g and 50% Reduction in UP:C Ratio From Baseline) [file jamanetwopen-e2228701-s001.pdf]

Supplemental Online Content

Gipson DS, Troost JP, Spino C, et al. Comparing kidney health outcomes in children, adolescents, and adults with focal segmental glomerulosclerosis. *JAMA Netw Open*. 2022;5(8):e2228701. doi:10.1001/jamanetworkopen.2022.28701

eTable 1. Minimal Detectable Differences by Outcome With 80% Power and 2-Sided Alpha

eTable 2. NEPTUNE: Baseline Demographic, Clinical, Genetic, and Laboratory Characteristics of Participants With FSGS by Age

eTable 3. FSGS-CT: Baseline Demographic, Clinical, Genetic, and Laboratory Characteristics of Randomized Participants by Age

eTable 4. KRN: Baseline Demographic, Clinical, and Laboratory Characteristics of Participants by Age at FSGS Biopsy

eTable 5. Pooled (NEPTUNE, KRN): Medication Exposures Before and After Biopsy by Age

eTable 6. NEPTUNE: Logistic Regression Models of Immunosuppressive Therapy Treatment (Treated vs Untreated) Among Participants Who Were Treatment Naive at Biopsy

eTable 7. Sensitivity Analyses of Potential Confounding and Excluding Monogenic Disease

eTable 8. Tests of Effect Modification in Cox Proportional Hazard Models of Time to ESKD or 40% Reduction in eGFR and of Time to Complete Remission by Study

eTable 9. Results of Linear-Mixed Effects Models of eGFR After Biopsy by Study and Pooled

eFigure 1. NEPTUNE: Participants Included in NEPTUNE Analyses

eFigure 2. KRN: Participants Included in KRN Analyses

eFigure 3. Time to ESKD by Age

eFigure 4. KRN: Time to ESKD by Age

eFigure 5. Time to ESKD or 40% Reduction in eGFR by Age: Significant Effect Modification by Sex in FSGS-CT

eFigure 6. Time to ESKD or 40% Reduction in eGFR by Age: Significant Effect Modification by Baseline UP:C Ratio in NEPTUNE

eFigure 7. Time To Complete or Partial Remission by Age (Partial Remission Defined as UP:C Ratio <3.5 g/g and 50% Reduction in UP:C Ratio From Baseline)

This supplemental material has been provided by the authors to give readers additional information about their work.

© 2022 Gipson DS et al. *JAMA Network Open.*

eTable 1. Minimal Detectable Differences by Outcome With 80% Power and 2-Sided Alpha

Outcome	Study	Comparison (vs. adults)	Observed difference*	Minimum Detectable Difference with 80% power*
			HR	HR
ESKD/40%reduction in eGFR	NEPTUNE	Children	0.95	0.38
		Adolescents	0.75	0.39
	FSGS-CT	Children	0.84	0.38
		Adolescents	0.92	0.39
	KRN	Children	1.24	0.55
		Adolescents	1.14	0.45
	Pooled	Children	1.12	0.62
		Adolescents	1.06	0.64
ESKD	NEPTUNE	Children	0.00	0.20
		Adolescents	0.00	0.21
	FSGS-CT	Children	0.53	0.26
		Adolescents	1.01	0.27
	KRN	Children	0.80	0.42
		Adolescents	1.01	0.52
	Pooled	Children	0.67	0.56
		Adolescents	0.85	0.58
			Mean Difference	Mean Difference
eGFR slope	NEPTUNE	Children	-2.12	5.92
		Adolescents	-2.14	5.70
	FSGS-CT	Children	-2.24	6.19
		Adolescents	-3.65	5.99
	KRN	Children	-0.61	6.28
		Adolescents	-0.85	4.74
	Pooled	Children	-1.61	3.29
		Adolescents	-2.13	3.05
Complete remission	NEPTUNE	Children	1.90	2.92
		Adolescents	1.88	2.81
	FSGS-CT	Children	1.77	3.32
		Adolescents	1.50	3.19

© 2022 Gipson DS et al. *JAMA Network Open.*

Outcome	Study	Comparison (vs. adults)	Observed difference*	Minimum Detectable Difference with 80% power*
	Pooled	Children	1.84	2.33
		Adolescents	1.68	2.10
Complete/partial remission	NEPTUNE	Children	1.06	2.06
		Adolescents	1.04	2.01
	FSGS-CT	Children	0.91	2.12
		Adolescents	0.90	2.07
	Pooled	Children	1.00	1.63
		Adolescents	1.00	1.62

*Hazard ratio for ESKD/40%reduction in eGFR, ESKD, complete remission, and complete/partial remission; mean difference in slope per year for eGFR slope

© 2022 Gipson DS et al. *JAMA Network Open.*

eTable 2. NEPTUNE: Baseline Demographic, Clinical, Genetic, and Laboratory Characteristics of Participants With FSGS by Age

Descriptive statistics are presented as number (percent) or median (25th, 75th percentile).

Characteristic	Descriptive Statistics				*p*-value comparisons				
	Overall (n=166)	1: Children Age<13 (n=32)	2: Adolescents Age:13- 17 (n=29)	3: Adults Age: ≥18 (n=105)	1 vs. 2 vs. 3	1+2 vs. 3	1 vs. 2	2 vs. 3	1 vs. 3
Age^, years	30 (14, 50)	7 (5, 11)	15 (14, 16)	46 (31, 58)	---	---	---	---	---
Female	67 (40)	19 (59)	9 (31)	39 (37)	0.04	0.27	0.03	0.54	0.03
Race					0.004	0.01	0.33	0.01	0.03
Asian	10 (6)	2 (7)	2 (7)	6 (6)					
Black	60 (37)	12 (41)	17 (63)	31 (30)					
Other	4 (2)	3 (10)	0 (0)	1 (1)					
White	85 (54)	12 (41)	8 (30)	65 (63)					
Unknown, n	7	3	2	2					
Hispanic	36 (22)	8 (25)	6 (22)	22 (21)	0.89	0.68	0.80	0.89	0.63
Age at onset	26 (12, 43)	5 (2, 10)	14 (13, 15)	41 (29, 58)	---	---	---	---	---
APOL1 genotype					<0.001	<0.001	0.04	<0.001	0.19
2 risk alleles	30 (20)	5 (22)	14 (50)	11 (11)					
0-1 risk alleles	117 (80)	18 (78)	14 (50)	85 (89)					
Unknown, n	19	9	1	9					
Interstitial fibrosis, %	15 (3, 27)	1 (0, 5)	10 (0, 18)	20 (10, 42)	<0.001	<0.001	0.01	<0.001	<0.001
Global sclerosis, %	10 (0, 39)	0 (0, 1)	7 (2, 14)	22 (7, 53)	<0.001	<0.001	0.004	0.007	<0.001
Segmental sclerosis, %	7 (3, 16)	4 (0, 13)	9 (3, 16)	8 (4, 18)	0.12	0.11	0.16	0.71	0.04
Edema, %	63 (39)	14 (44)	11 (42)	38 (37)	0.76	0.47	0.91	0.64	0.51
Missing	0	0	3	3					
Blood pressure^					<0.001	<0.001	0.01	<0.001	0.002
Normal	60 (38)	10 (32)	19 (70)	31 (30)					
Pre-hypertensive	50 (31)	4 (13)	1 (4)	45 (44)					
Hypertension	50 (31)	17 (55)	7 (26)	26 (25)					
Unknown, n	6	1	2	3					
Weight status^					0.60	0.44	0.04	0.02	0.96
Underweight	3 (2)	1 (3)	0 (0)	2 (2)					
Normal weight	35 (23)	5 (16)	12 (46)	18 (19)					

© 2022 Gipson DS et al. *JAMA Network Open.*

Overweight	45 (29)	11 (34)	3 (12)	31 (32)					
Obese	72 (46)	15 (47)	11 (42)	46 (47)					
**Characteristic**	**Descriptive Statistics**				***p*-value comparisons**				
Unknown, n	11	0	3	8					
UP:C^ (g/g)	3.8 (1.9, 7.5)	6.4 (3.1, 15.1)	2.3 (1.3, 7.9)	3.7 (1.9, 5.7)	0.003	0.13	0.005	0.38	0.002
0.3-1.4	29 (17)	4 (13)	8 (28)	17 (16)					
1.5-3.4	50 (30)	5 (16)	11 (38)	34 (32)					
≥3.5	87 (52)	23 (72)	10 (34)	54 (51)					
eGFR^, ml/min/1.73m^2	72 (46, 95)	94 (79, 113)	80 (69, 95)	57 (36, 85)	<0.001	<0.001	0.02	0.002	<0.001
≥90	49 (30)	18 (58)	9 (35)	22 (21)					
60-89	51 (31)	12 (39)	14 (5654	25 (24)					
30-59	48 (30)	1 (3)	3 (12)	44 (42)					
≤30	14 (9)	0 (0)	0 (0)	14 (13)					
Unknown, n	4	1	0	0					
Serum albumin^, g/dL	3.3 (2.4, 4.0)	3.1 (2.0, 3.7)	3.1 (2.2, 4.2)	3.7 (2.8, 4.1)	0.02	0.02	0.33	0.52	0.004
≥3.5	53 (46)	8 (28)	10 (48)	35 (53)					
2.5-3.4	31 (27)	9 (31)	2 (10)	20 (30)					
<2.5	32 (28)	12 (41)	9 (43)	11 (17)					
Unknown, n	50	3	8	39					
Nephrotic syndrome	54 (33)	18 (56)	8 (28)	28 (27)	0.01	0.03	0.02	0.92	0.002
Treatment before biopsy									
Steroids	42 (25)	24 (75)	8 (28)	10 (10)	<0.001	<0.001	<0.001	0.01	<0.001
CNI	9 (5)	3 (9)	3 (10)	3 (3)	0.16	0.06	0.90	0.08	0.11
CTX	2 (1)	1 (3)	0 (0)	1 (1)	0.50	0.70	0.34	0.60	0.37
MMF	6 (4)	4 (13)	1 (3)	1 (1)	0.01	0.02	0.20	0.33	0.002
Rituximab	0 (0)	0 (0)	0 (0)	0 (0)	---	---	---	---	---
Other IST	3 (2)	0 (0)	0 (0)	3 (3)	0.41	0.18	---	0.36	0.33
RAAS Blockade	71 (43)	11 (34)	7 (24)	53 (50)	0.02	0.01	0.38	0.01	0.11

^ at baseline

Abbreviations: *APOL1*, Apolipoprotein L1; CNI, Calcineurin inhibitors; CTX, Cytoxan; eGFR, estimated glomerular filtration rate; HDL, high density lipoprotein; IQR, interquartile range; IST, immunosuppressive therapy; LDL, low density lipoprotein; MMF, mycophenolate mofetil; NEPTUNE, Nephrotic Syndrome Study Network; RAAS, renin-angiotensin system; UP:C, urine protein: creatinine ratio

Definitions: *p*-value comparisons are from Kreskas-Wallis tests for continuous variables and chi-square tests for categorical variables. Percentages and *p*-values are based on complete case analyses (i.e., do not include participants with missing data). eGFR was calculated using the creatinine-based modified CKiD formula in children and adolescents and the CKD-EPI equation in adults.[17, 18] Blood pressure categorized as follows: normal = systolic<=120 mm Hg and diastolic<=8 mm Hg 0, or systolic and diastolic percentile<=90 for

© 2022 Gipson DS et al. *JAMA Network Open.*

children and adolescents; Pre-hypertensive(systolic>120 mm Hg or diastolic>80 mm Hg,or systolic/diastolic percentile 90-95 for children and adolescents; Hypertension= systolic>140 mm Hg or diastolic >90 mm Hg, or systolic/diastolic percentile>95 for children and adolescents.[12, 13] Weight status categorized as follows: underweight=BMI<18.5 or BMI percentile <5 for children and adolescents; normal weight=BMI18.5-25 or BMI percentile 5-85 for children and adolescents; overweight=BMI 25-30 of BMI percentile 85-95 for children and adolescents; obese=BMI≥30 or BMI percentile >95th for children and adolescents.[14] Nephrotic Syndrome is categorized as UP:C≥3.5 + edema or serum albumin <3.0.

© 2022 Gipson DS et al. *JAMA Network Open.*

eTable 3. FSGS-CT: Baseline Demographic, Clinical, Genetic, and Laboratory Characteristics of Randomized Participants by Age

Descriptive statistics are presented as number (percent) or median (25th, 75th percentile).

Characteristic	Descriptive Statistics				*p*-value comparisons				
	Overall (n=132)	1: Children Age<13 (n=42)	2: Adolescents Age:13- 17 (n=48)	3: Adults Age: ≥18 (n=42)	1 vs. 2 vs. 3	1+2 vs. 3	1 vs. 2	2 vs. 3	1 vs. 3
Age^, years	15 (11, 23)	7 (4, 11)	15 (14, 16)	31 (23, 34)	---	---	---	---	---
Female	61 (46)	19 (45)	22 (46)	20 (75)	0.97	0.82	0.95	0.87	0.99
Race					0.32	0.44	0.34	0.33	0.34
Asian	3 (2)	2 (5)	1 (2)	0 (0)					
Black	50 (38)	15 (36)	16 (33)	19 (45)					
Native American/ Pacific Islander	2 (1)	1 (2)	0 (0)	1 (2)					
Other	2 (1)	2 (5)	0 (0)	0 (0)					
White	75 (57)	22 (52)	31 (65)	22 (52)					
Hispanic	23 (17)	6 (14)	12 (26)	5 (12)	0.21	0.25	0.20	0.11	0.75
Age at onset, years	14 (9, 22)	6 (3, 10)	14 (13, 15)	29 (23, 33)	---	---	---	---	---
APOL1 genotype					0.01	0.02	0.03	0.33	0.003
2 risk alleles	25 (23)	2 (6)	10 (25)	13 (35)					
0-1 risk alleles	86 (77)	32 (94)	30 (75)	24 (65)					
Unknown, n	21	8	8	5					
Interstitial fibrosis, %	10 (4, 25)	5 (0, 10)	10 (5, 20)	18 (5, 40)	<0.001	<0.001	0.001	0.03	<0.001
Global sclerosis, %	0 (0, 12)	0 (0, 8)	0 (0, 11)	4 (0, 25)	0.14	0.06	0.59	0.19	0.05
Segmental sclerosis, %	20 (9, 33)	11 (6, 20)	25 (10, 38)	22 (11, 36)	0.001	0.11	<0.001	0.73	0.001
Edema					0.20	0.03	0.85	0.11	0.05
None	76 (58)	25 (60)	27 (56)	24 (57)					
Pretibial	38 (29)	10 (23)	13 (27)	16 (36)					
Above knee	2 (2)	0 (0)	1 (2)	1 (2)					
Presacral	2 (1)	0 (0)	0 (0)	2 (5)					
Ascites	9 (7)	4 (9)	5 (10)	0 (0)					
Anasarca	5 (4)	3 (7)	2 (4)	0 (0)					
Blood pressure^					<0.001	0.02	0.001	0.09	0.002
Normal	53 (40)	12 (29)	27 (56)	14 (34)					
Pre-hypertensive	36 (27)	6 (14)	12 (25)	18 (44)					

© 2022 Gipson DS et al. *JAMA Network Open.*

Hypertension	42 (32)	24 (57)	9 (18)	9 (22)					
Unknown, n	1	0	0	1					
**Characteristic**	**Descriptive Statistics**				***p*-value comparisons**				
Weight status^					0.12	0.28	0.08	0.06	0.67
Underweight	0 (0)	0 (0)	0 (0)	0 (0)					
Normal weight	50 (38)	15 (36)	23 (48)	12 (29)					
Overweight	33 (25)	8 (19)	14 (29)	11 (26)					
Obese	49 (37)	19 (45)	12 (23)	19 (45)					
UP:C at enrollment, g/g	4.0 (2.2, 8.4)	5.8 (2.9, 8.5)	4.3 (2.7, 10.3)	3.0 (1.7, 5.1)	0.01	0.007	0.31	0.05	0.005
0.3-1.4	21 (16)	5 (12)	7 (15)	9 (21)					
1.5-3.4	37 (28)	8 (19)	13 (27)	16 (38)					
≥3.5	74 (56)	29 (69)	28 (58)	17 (40)					
eGFR^, ml/min/1.73m^2	82 (60, 116)	123 (78, 154)	79 (55, 108)	68 (49, 96)	<0.001	<0.001	<0.001	0.27	<0.001
≥90	59 (45)	29 (69)	20 (42)	10 (24)					
60-89	36 (27)	7 (17)	13 (27)	16 (38)					
30-59	34 (26)	5 (12)	13 (27)	16 (38)					
≤30	3 (2)	1 (2)	2 (4)	0 (0)					
Serum albumin^, g/dL	2.8 (2.1, 3.7)	2.4 (1.8, 2.9)	2.8 (2.1, 3.8)	3.6 (2.5, 3.9)	0.001	0.001	0.07	0.09	0.001
≥3.5	48 (33)	5 (12)	16 (33)	21 (50)					
2.5-3.4	42 (32)	15 (35)	15 (31)	12 (29)					
<2.5	42 (32)	22 (52)	17 (35)	9 (21)					
Nephrotic syndrome	60 (45)	28 (67)	20 (42)	12 (29)	0.002	0.01	0.02	0.20	<0.001
Steroids before enrollment, months	3 (2, 5)	3 (2, 6)	2 (1, 4)	4 (2, 6)	0.02	0.07	0.04	0.008	0.73

Abbreviations: *APOL1*, Apolipoprotein L1; CSA, Cyclosporine; eGFR, estimated glomerular filtration rate; IQR, interquartile range; IST, immunosuppressive therapy; MMF, mycophenolate mofetil; UP:C, urine protein: creatinine ratio

^ at baseline

Definitions: *p*-value comparisons are from Kruskal-Wallis tests for continuous variables and chi-square tests for categorical variables. Percentages and *p*-values are based on complete case analyses (i.e., do not include participants with missing data). eGFR was calculated using the creatinine-based modified CKiD formula in children and adolescents and the CKD-EPI equation in adults.[17, 18] Blood pressure categorized as follows: normal = systolic<=120 mm Hg and diastolic<=8 mm Hg 0, or systolic and diastolic percentile<=90 for children and adolescents; Pre-hypertensive(systolic>120 mm Hg or diastolic>80 mm Hg,or systolic/diastolic percentile 90-95 for children and adolescents; Hypertension= systolic>140 mm Hg or diastolic >90 mm Hg, or systolic/diastolic percentile>95 for children and adolescents.[12, 13] Weight status categorized as follows: underweight=BMI<18.5 or BMI percentile <5 for children and adolescents; normal weight=BMI18.5-25 or BMI percentile 5-85 for children and adolescents; overweight=BMI 25-30 of BMI percentile 85-95 for children and adolescents; obese=BMI≥30 or BMI percentile >95th for children and adolescents.[14] Nephrotic Syndrome is categorized as UP:C≥3.5 + edema or serum albumin <3.0.

eTable 4. KRN: Baseline Demographic, Clinical, and Laboratory Characteristics of Participants by Age at FSGS Biopsy

Descriptive statistics are presented as number (percent) or median (25th, 75th percentile).

**Characteristic**	**Descriptive Statistics**				***p*-value comparisons**				
	Overall (n=184)	1: Children Age<13 (n=53)	2: Adolescents Age:13- 17 (n=25)	3: Adults Age: ≥18 (n=106)	1 vs. 2 vs. 3	1+2 vs. 3	1 vs. 2	2 vs. 3	1 vs. 3
Age at biopsy, years	22 (9, 42)	6 (3, 8)	15 (13, 17)	39 (28, 53)	---	---	---	---	---
Female	87 (47)	23 (43)	10 (40)	54 (51)	0.49	0.25	0.78	0.32	0.37
Race					0.10	0.12	0.20	0.29	0.08
Asian	16 (9	8 (15)	0 (0)	8 (8)					
Black	28 (15)	8 (15)	5 (20)	15 (14)					
Other	21 (11)	2 (4)	2 (8)	17 (16)					
White	115 (63)	35 (66)	18 (72)	62 (58)					
Unknown, n	4 (2)	0 (0)	0 (0)	4 (4)					
Hispanic	39 (21)	7 (13)	9 (36)	23 (22)	0.07	0.74	0.02	0.17	0.16
Age at kidney disease onset, years	20 (8, 41)	3 (2, 6)	13 (12, 16)	39 (26, 53)	---	---	---	---	---
UP:C within 1 month of biopsy, g/g	4.0 (1.7, 7.6)	3.6 (0.8, 10.1)	2.4 (2.0, 5.3)	4.2 (1.9, 7.7)	0.64	0.44	0.71	0.32	0.70
eGFR within 1 month of biopsy, ml/min/1.73m²	64 (33, 105)	138 (98, 170)	94 (57, 118)	42 (29, 90)	<0.001	<0.001	0.03	0.01	<0.001
Serum albumin within 1 month of biopsy, g/dL	3.2 (2.0, 3.8)	3.0 (1.6, 3.5)	3.3 (2.9, 3.4)	3.6 (2.3, 3.9)	0.27	0.14	0.39	0.43	0.13
Treatment before biopsy									
Steroids	38 (21)	17 (32)	4 (16)	17 (16)	0.05	0.07	0.14	0.99	0.02
CNI	21 (11)	8 (15)	3 (12)	10 (9)	0.57	0.33	0.71	0.70	0.29
CTX	8 (4)	5 (9)	2 (8)	1 (1)	0.03	0.01	0.84	0.03	0.01

© 2022 Gipson DS et al. *JAMA Network Open.*

MMF	15 (8)	4 (8)	3 (12)	8 (8)	0.75	0.73	0.52	0.47	1.00
Rituximab	3(2)	0(0)	0(0)	3(3)	0.33	0.13	---	0.39	0.22
**Characteristic**	**Descriptive Statistics**				***p*-value comparisons**				
RAAS Blockade	29 (16)	6(11)	3(12)	20(19)	0.04	0.18	0.93	0.42	0.23
IST after biopsy (among those naïve at biopsy)									
Steroids	59 (40)	15 (42)	6 (29)	38 (43)	0.49	0.48	0.32	0.23	0.92
CNI	55 (34)	22 (49)	4 (18)	29 (30)	0.02	0.25	0.02	0.26	0.03
CTX	4 (2)	1 (2)	0 (0)	3 (3)	0.70	0.53	0.49	0.41	0.78
MMF	39 (23)	13 (27)	5 (23)	21 (21)	0.79	0.55	0.73	0.89	0.49
Rituximab	24 (13)	14 (26)	2 (8)	8 (8)	0.004	0.01	0.06	0.97	0.002
RAAS Blockade after biopsy (among those naïve at biopsy)	83 (54)	20 (43)	10 (45)	53 (62)	0.08	0.02	0.82	0.17	0.03

Abbreviations: CNI, Calcineurin inhibitors; CTX, Cytoxan; eGFR, estimated glomerular filtration rate; IQR, interquartile range; IST, immunosuppressive therapy; KRN, Kidney Research Network; MMF, mycophenolate mofetil; RAAS, renin-angiotensin system; UP:C, urine protein: creatinine ratio

Definitions: *p*-value comparisons are from Kruskal-Wallis tests for continuous variables and chi-square tests for categorical variables. Percentages and *p*-values are based on complete case analyses (i.e., do not include participants with missing data). eGFR was calculated using the creatinine-based modified CKiD formula in children and adolescents and the CKD-EPI equation in adults.[17, 18]

© 2022 Gipson DS et al. *JAMA Network Open.*

eTable 5. Pooled (NEPTUNE, KRN): Medication Exposures Before and After Biopsy by Age

Descriptive statistics are presented as number (percent)

Characteristic	Descriptive Statistics				*p*-value comparisons				
	Overall (n=350)	1: Children Age<13 (n=85)	2: Adolescents Age:13- 17 (n=54)	3: Adults Age: ≥18 (n=211)	1 vs. 2 vs. 3	1+2 vs. 3	1 vs. 2	2 vs. 3	1 vs. 3
IST before biopsy									
Steroids	80 (23)	41 (48)	12 (22)	27 (13)	<0.001	<0.001	0.002	0.08	<0.001
CNI	30 (9)	11 (13)	6 (11)	13 (6)	0.13	0.05	0.75	0.21	0.05
CTX	10 (3)	6 (7)	2 (4)	2 (1)	0.02	0.01	0.41	0.14	0.003
MMF	21 (6)	8 (9)	4 (7)	9 (4)	0.22	0.09	0.68	0.34	0.09
Rituximab	(1)	0 (0)	0 (0)	3 (1)	0.37	0.16	---	0.38	0.27
RAAS Blockade	100 (29)	17 (20)	10 (19)	73 (35)	0.01	0.002	0.83	0.02	0.01
IST after biopsy (among those naïve at biopsy)									
Steroids	115 (43)	21 (48)	15 (36)	79 (43)	0.52	0.87	0.26	0.39	0.56
CNI	97 (30)	40 (54)	18 (38)	39 (20)	<0.001	<0.001	0.07	0.01	<0.001
CTX	8 (2)	3 (4)	0 (0)	5 (2)	0.37	0.95	0.16	0.26	0.52
MMF	51 (16)	19 (25)	7 (14)	25 (12)	0.04	0.05	0.15	0.76	0.01
Rituximab	29 (8)	17 (20)	3 (6)	9 (4)	<0.001	0.001	0.02	0.70	<0.001
RAAS Blockade after biopsy (among those naïve at biopsy)	155 (62)	34 (50)	30 (68)	91 (66)	0.06	0.15	0.06	0.78	0.03

Abbreviations: CNI, Calcineurin inhibitors; CTX, Cytoxan; IST, immunosuppressive therapy; KRN, Kidney Research Network; MMF, mycophenolate mofetil; RAAS, renin-angiotensin system.

Definitions: *p*-value comparisons are from Kruskal-Wallis tests for continuous variables and chi-square tests for categorical variables. Percentages and *p*-values are based on complete case analyses (i.e., do not include participants with missing data).

© 2022 Gipson DS et al. *JAMA Network Open.*

eTable 6. NEPTUNE: Logistic Regression Models of Immunosuppressive Therapy Treatment (Treated vs Untreated) Among Participants Who Were Treatment Naive at Biopsy

(n=117 participants; n=57 events)

**Unadjusted model**	**Odds ratio**	**95% confidence interval**	***P-value***
Age, years			
Children (<13)	3.95	0.76 to 20.59	0.10
Adolescents (13-17)	1.76	0.68 to 4.56	0.25
Adults (≥18)	REF	REF	REF
UP:C (g/g) at enrollment			
0.3-1.4	0.09	0.03 to 0.28	<0.001
1.5-3.4	0.11	0.04 to 0.28	<0.001
≥3.5	REF	REF	REF
eGFR (per 30ml/min/1.73m^2)	1.08	0.73 to 1.59	0.70
**Adjusted model**			
Age, years			
Children (<13)	4.19	0.50 to 35.29	0.19
Adolescents (13-17)	6.84	1.80 to 25.96	0.005
Adults (≥18)	REF	REF	REF
UP:C at enrollment, g/g			
0.3-1.4	0.04	9.8E-03 to 0.16	<0.001
1.5-3.4	0.07	0.03 to 0.22	<0.001
≥3.5	REF	REF	REF
eGFR (per 30ml/min/1.73m^2)	1.08	0.65 to 1.78	0.77

Abbreviations: eGFR, estimated glomerular filtration rate; IST, immunosuppressive therapy; NEPTUNE, Nephrotic Syndrome Study Network; REF, reference; UP:C, urine protein: creatinine ratio;

© 2022 Gipson DS et al. *JAMA Network Open.*

eTable 7. Sensitivity Analyses of Potential Confounding and Excluding Monogenic Disease

	**NEPTUNE**		**FSGS-CT**		**KRN**
	Known monogenic excluded	Known monogenic included	Known monogenic excluded	Known monogenic included	
Adjusted HR time to kidney failure					
Child vs. adult	2.63 [0.75, 9.27]	2.68 [0.77, 9.37]	1.31 [0.52, 3.28]	1.29 [0.55, 3.02]	1.02 [0.56, 1.85]
Adolescent vs. adult	2.09 [0.67, 6.51]	1.99 [0.64, 6.20]	1.44 [0.68, 3.06]	1.23 [0.60, 2.50]	0.92 [0.47, 1.80]
Adjusted difference in eGFR slope					
Child vs. adult	-2.25 [-6.33, 1.83]	-2.03 [-5.92, 1.86]	-2.19 [-9.15, 4.76]	-2.30 [-9.06, 4.45]	0.02 [-0.58, 0.61]
Adolescent vs. adult	-3.36 [-7.32, 0.60]	-2.89 [-6.68, 0.90]	-5.86 [-12.76, 1.04]	-5.23 [-11.85, 1.38]	-0.09 [-0.76, 0.59]
Adjusted HR time to remission					
Child vs. adult	0.85 [0.30, 2.37]	0.81 [0.30, 2.19]	2.08 [0.83, 5.21]	1.87 [0.76, 4.58]	---
Adolescent vs. adult	1.51 [0.58, 3.92]	1.54 [0.60, 3.97]	1.30 [0.57, 2.96]	1.18 [0.53, 2.64]	---
Adjusted HR time to FPRE					
Child vs. adult	0.73 [0.34, 1.55]	0.57 [0.28, 1.18]	0.86 [0.42, 1.75]	0.85 [0.42, 1.73]	---
Adolescent vs. adult	0.72 [0.34, 1.55]	0.65 [0.31, 1.39]	1.13 [0.57, 2.21]	1.03 [0.54, 1.94]	---

NEPTUNE: Adjusted for interstitial fibrosis, global sclerosis, steroid treatment, APOL1, and baseline eGFR and UP:C
FSGS-CT: adjusted for interstitial fibrosis, segmental sclerosis, months of prior steroids, APOL1, and baseline eGFR and UP:C
KRN: adjusted for baseline eGFR and CNI treatment

© 2022 Gipson DS et al. *JAMA Network Open.*

eTable 8. Tests of Effect Modification in Cox Proportional Hazard Models of Time to ESKD or 40% Reduction in eGFR and of Time to Complete Remission by Study

Effect modifier	Time to ESKD or 40% reduction in eGFR , p-value^	Time to complete remission, p-value^
**NEPTUNE**		
Sex	0.10	0.78
Disease duration (<6 months vs. ≥6 months)	0.65	0.47
APOL1 genotype (2 vs. 0-1 risk alleles)	0.75	0.94
UP:C at enrollment (>3.5g/g vs. ≤3.5g/g)	0.008	0.58
Nephrotic Syndrome at enrollment	<0.001	0.13
Steroids (treated vs. untreated)	0.76	0.99
CNI (treated vs. untreated)	0.53	0.99
**FSGS-CT**		
Sex	0.03	0.17
APOL1 genotype (2 vs. 0-1 risk alleles)	0.35	0.99
UP:C at enrollment (>3.5g/g vs. ≤3.5g/g)	0.16	0.06
Nephrotic Syndrome at enrollment	0.09	0.11
Treatment arm: CNI vs. MMF+dexamethasone	0.82	0.21
**KRN**		
Sex	0.56	---
Disease duration (<6 months vs. ≥6 months)	0.25	---
Steroids (treated vs. untreated)	0.07	---
CNI (treated vs. untreated)	0.52	---
**Pooled**		
Sex	0.19	0.33
Disease duration (<6 months vs. ≥6 months)	0.19	0.08
APOL1 genotype (2 vs. 0-1 risk alleles)	0.66	0.64
UP:C at enrollment (>3.5g/g vs. ≤3.5g/g)	0.01	0.09
Nephrotic syndrome at enrollment	0.05	0.23
Steroids (treated vs. untreated)	0.15	---
CNI (treated vs. untreated)	0.21	---

Abbreviations: *APOL1*, Apolipoprotein L1; CNI, Calcineurin inhibitors; eGFR, estimated glomerular filtration rate; MMF, mycophenolate mofetil; NEPTUNE, Nephrotic Syndrome Study Network; UP:C, urine protein: creatinine ratio
Definitions: Nephrotic Syndrome is categorized as UP:C≥3.5 + edema or serum albumin <3.0.
^p-value based on test of the interaction of potential effect modifier and age in separate Cox proportional hazards models for each outcome

© 2022 Gipson DS et al. *JAMA Network Open.*

eTable 9. Results of Linear-Mixed Effects Models of eGFR After Biopsy by Study and Pooled

Covariate	Beta coefficient	95% confidence interval	*p-value*
**A: NEPTUNE (n=166 participants; n=1,827 observations)**			
Intercept	60.24	---	---
Time (years)	-2.19	-3.92 to -0.46	0.01
Child vs. adult	34.20	23.19 to 45.21	<0.001
Adolescent vs. adult	20.25	8.81 to 31.68	<0.001
Time (years)*Child vs. adult	-2.12	-5.54 to 1.29	0.22
Time (years)*Adolescent vs. adult	-2.14	-5.58 to 1.29	0.22
**B: FSGS-CT (n=132 participants; n=1,992 observations)**			
Intercept	63.61	---	---
Time (years)	-2.64	-6.96 to 1.69	0.23
Child vs. adult	30.88	20.08 to 41.68	<0.001
Adolescent vs. adult	10.79	0.39 to 21.20	0.04
Time (years)*Child vs. adult	-2.24	-8.28 to 3.80	0.47
Time (years)*Adolescent vs. adult	-3.65	-9.57 to 2.26	0.23
**C: KRN (n=184 participants; n=3,082 observations)**			
Intercept	55.74	---	---
Time (years)	-1.15	-4.31 to 2.01	0.47
Child vs. adult	37.67	21.14 to 54.19	<0.001
Adolescent vs. adult	21.80	2.11 to 41.49	0.03
Time (years)*Child vs. adult	-0.25	-4.14 to 3.65	0.90
Time (years)*Adolescent vs. adult	-0.61	-5.77 to 4.55	0.82
**D: Pooled (NEPTUNE + FSGS-CT + KRN) (n=482 participants; n=6,901 observations)**			
Intercept	60.59	---	---
Time (years)	-1.71	-3.23 to -0.19	0.03
Child vs. adult	33.69	27.04 to 40.34	<0.001
Adolescent vs. adult	15.91	9.06 to 22.76	<0.001
Time (years)*Child vs. adult	-1.61	-3.97 to 0.75	0.18
Time (years)*Adolescent vs. adult	-2.13	-4.66 to 0.40	0.10

NOTE: Results correspond to Figure 2.
Definitions: eGFR distribution was winsorized to account for hyperfiltration. All eGFR values over 120 ml/min/1.73m^2 were imputed to 120 ml/min/1.73m^2. Model included terms for random slope (time) and intercept (person).

© 2022 Gipson DS et al. *JAMA Network Open.*

eFigure 1. NEPTUNE: Participants Included in NEPTUNE Analyses

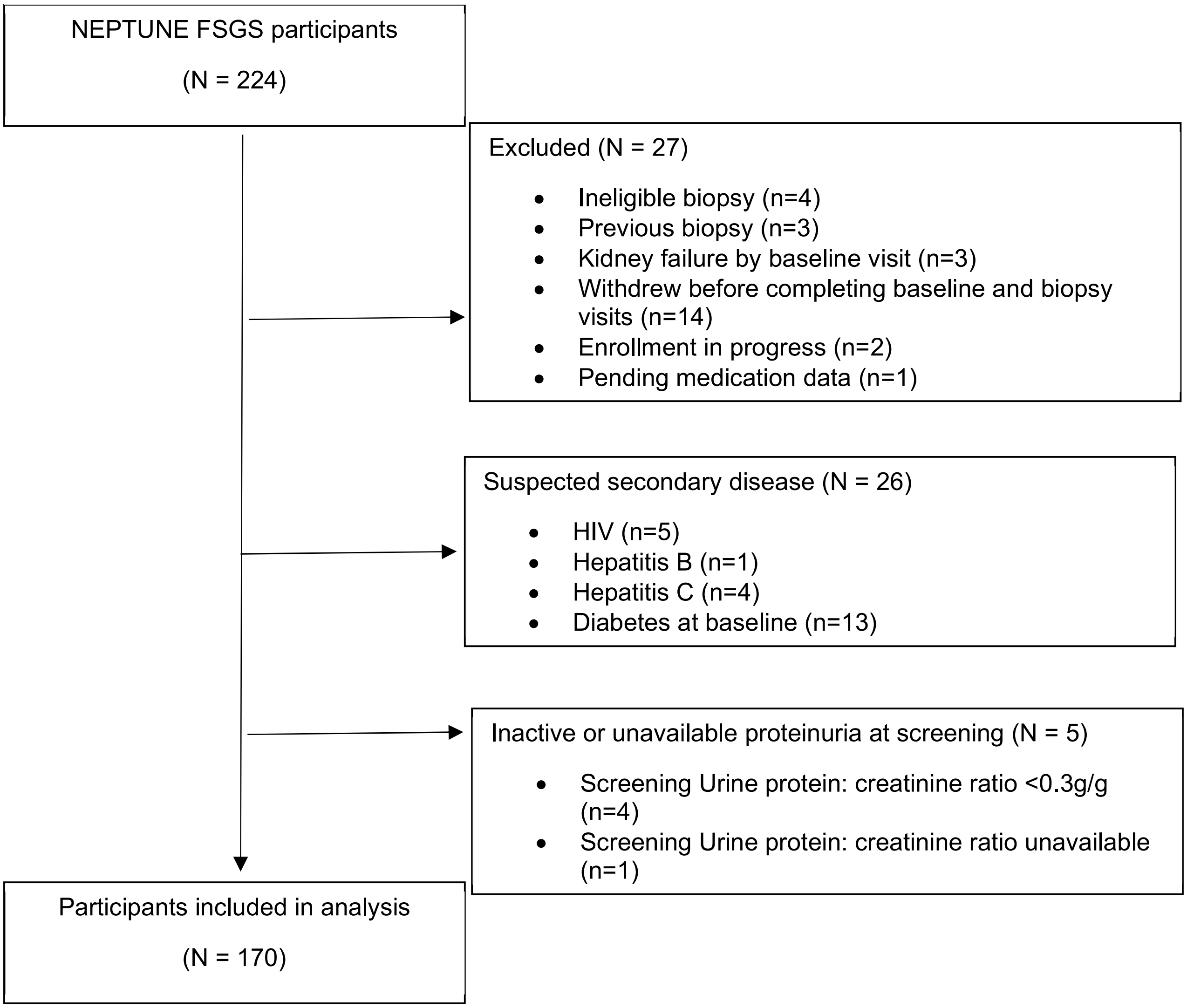

© 2022 Gipson DS et al. *JAMA Network Open.*

eFigure 2. KRN: Participants Included in KRN Analyses

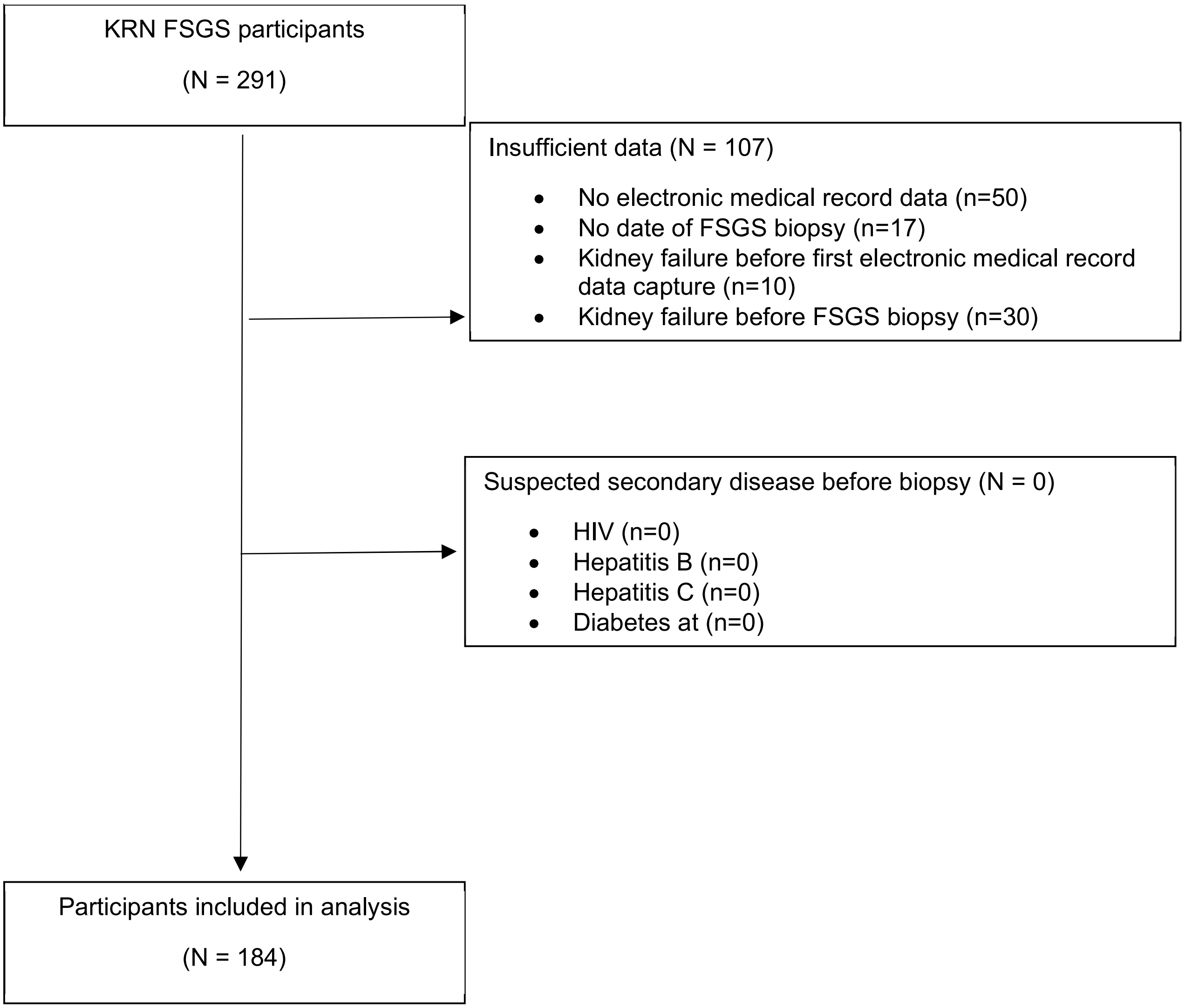

© 2022 Gipson DS et al. *JAMA Network Open.*

eFigure 3. Time to ESKD by Age

eFigure 3A. Neptune - Time to ESKD by age Kaplan-Meier curve with logrank test

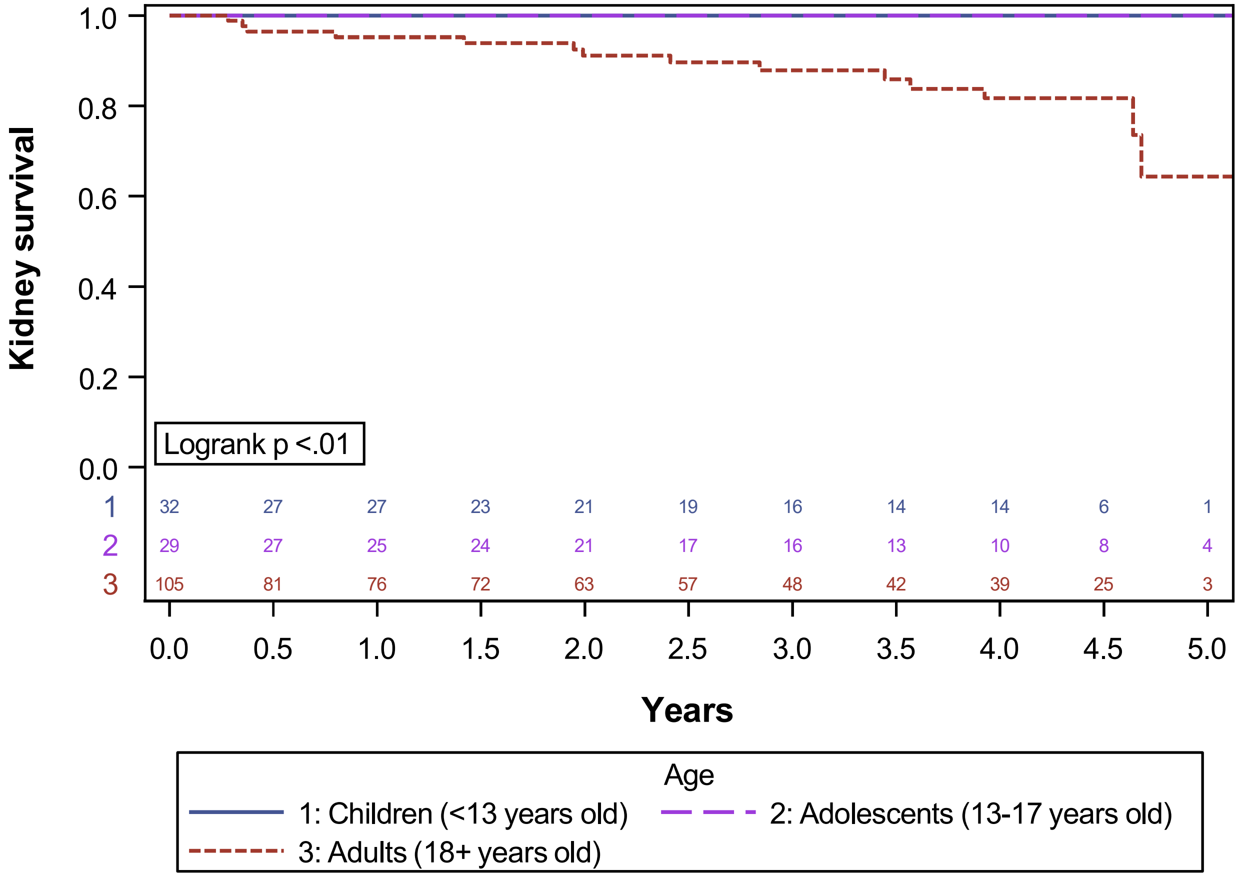

© 2022 Gipson DS et al. *JAMA Network Open.*

eFigure 4B. FSGS-CT - Time to ESKD by age Kaplan-Meier curve with logrank test

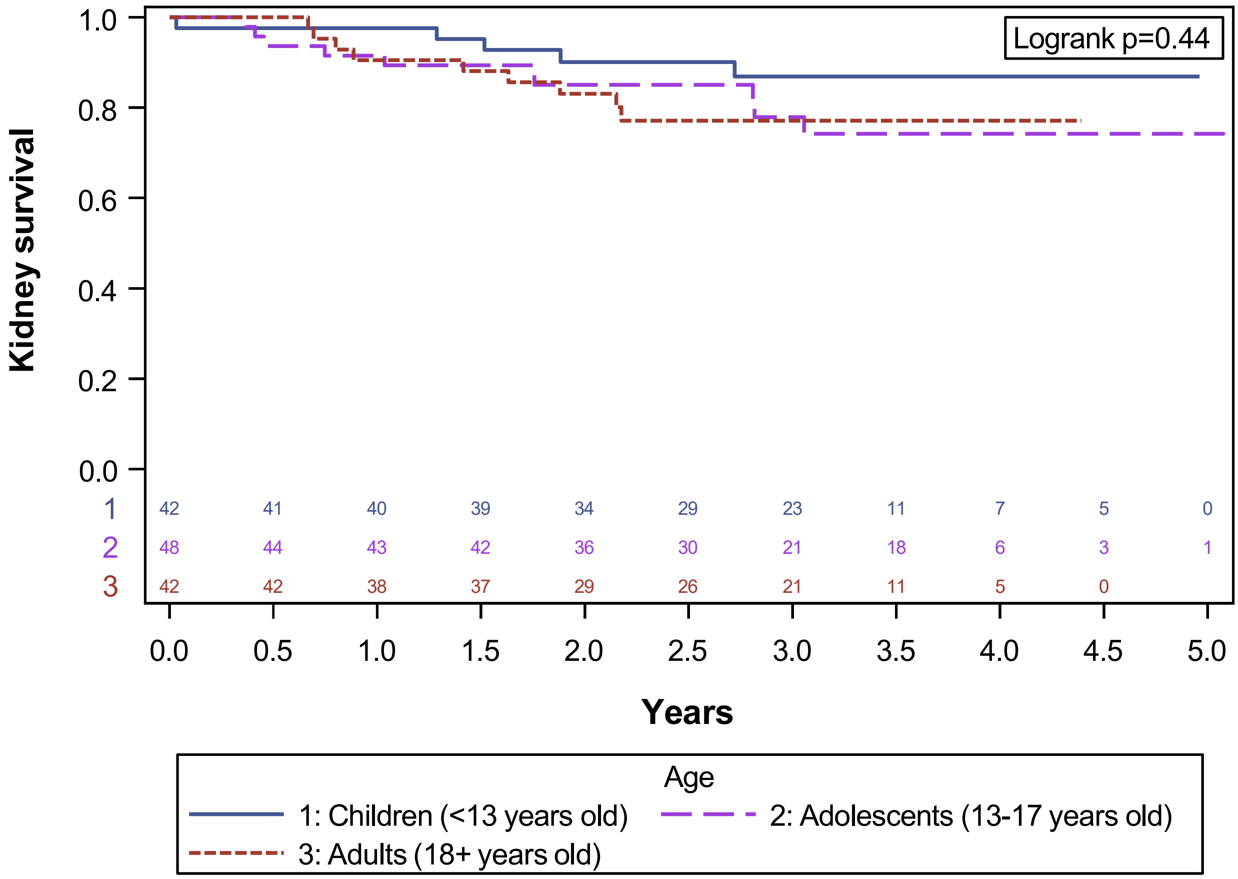

B: FSGS-CT n=132 participants; n=24 events

© 2022 Gipson DS et al. *JAMA Network Open.*

eFigure 3C. KRN - Time to ESKD by age Kaplan-Meier with logrank test

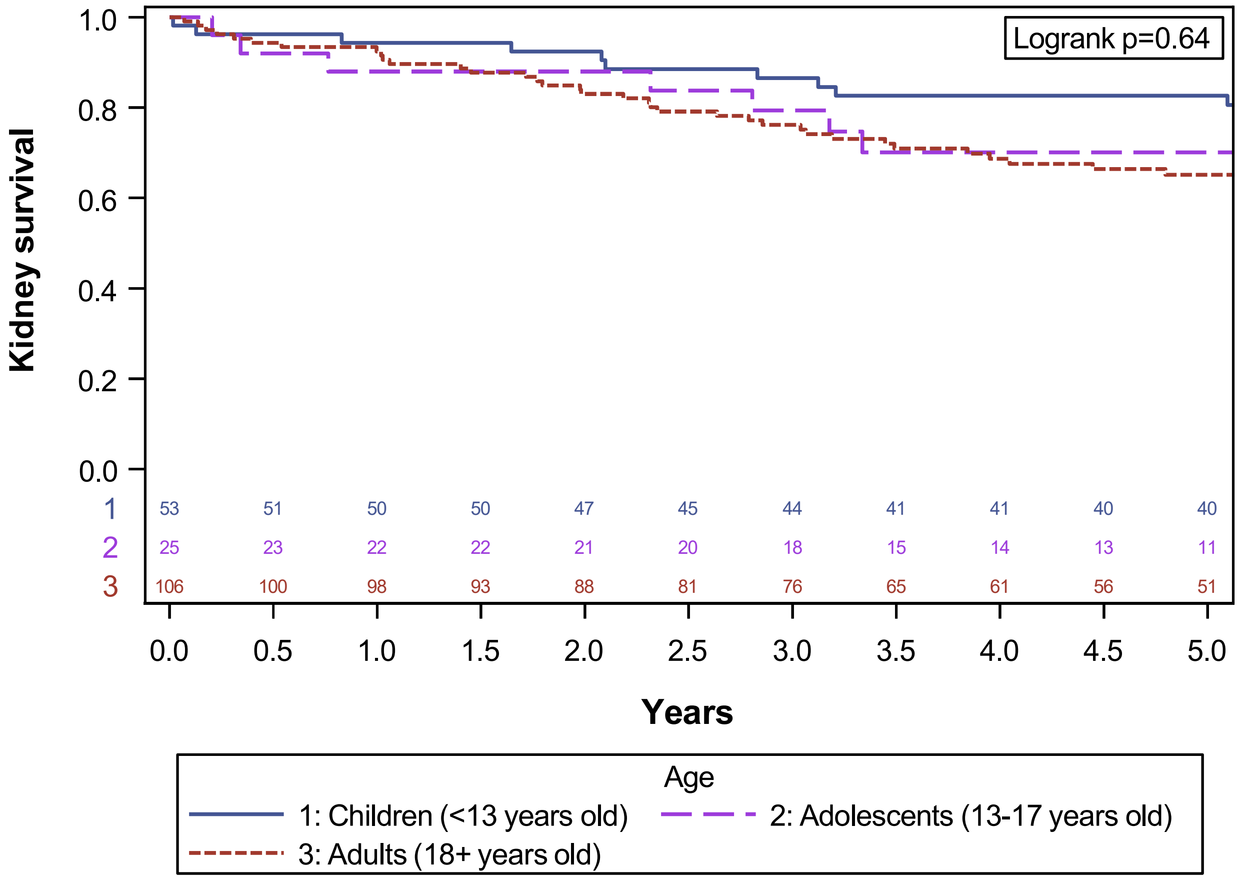

C: KRN n=184 participants; n=91 events

© 2022 Gipson DS et al. *JAMA Network Open.*

eFigure 3D. Pooled - Time to ESKD by age Kaplan-Meier with logrank test

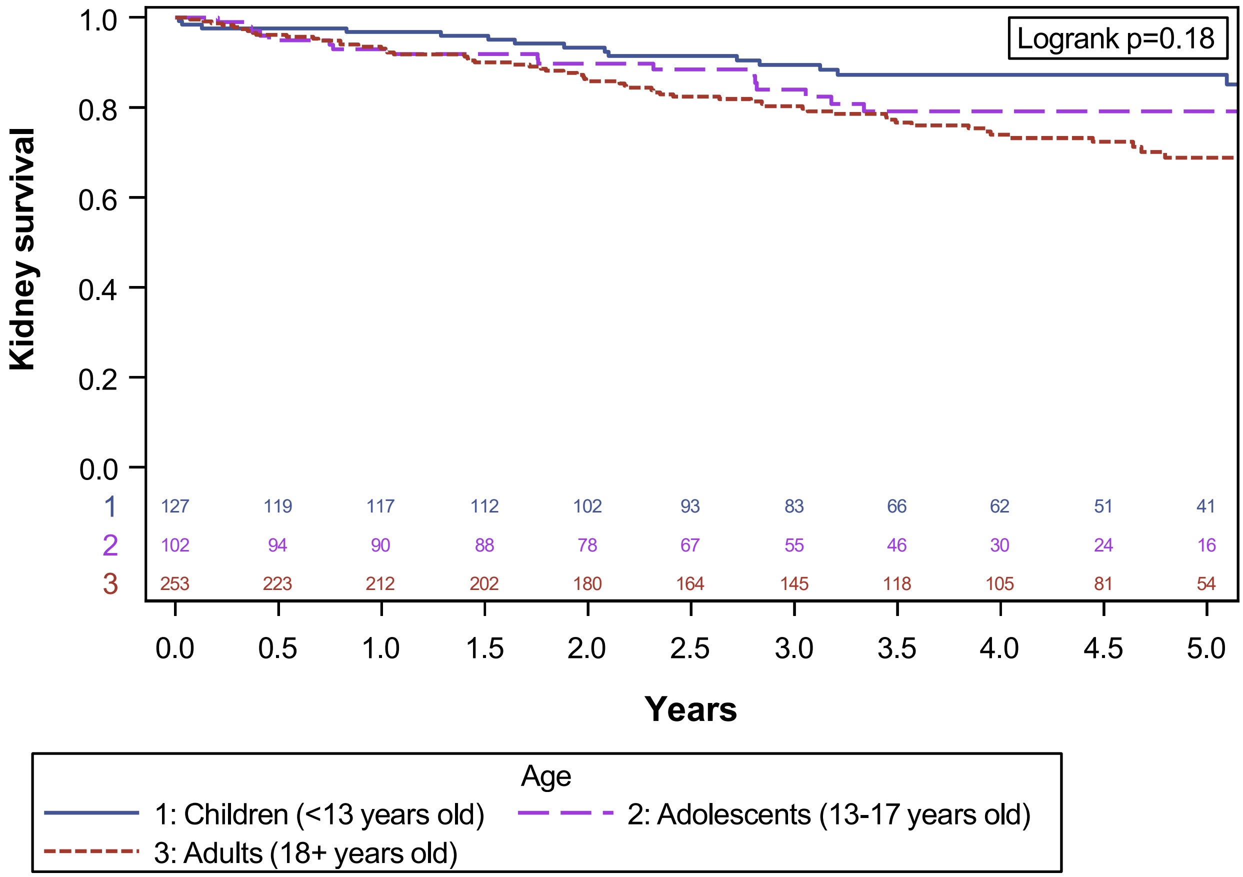

D: Pooled (NEPTUNE + FSGS-CT + KRN) n=482 participants; n=129 events

© 2022 Gipson DS et al. *JAMA Network Open.*

eFigure 4. KRN: Time to ESKD by Age

Kaplan-Meier with logrank test

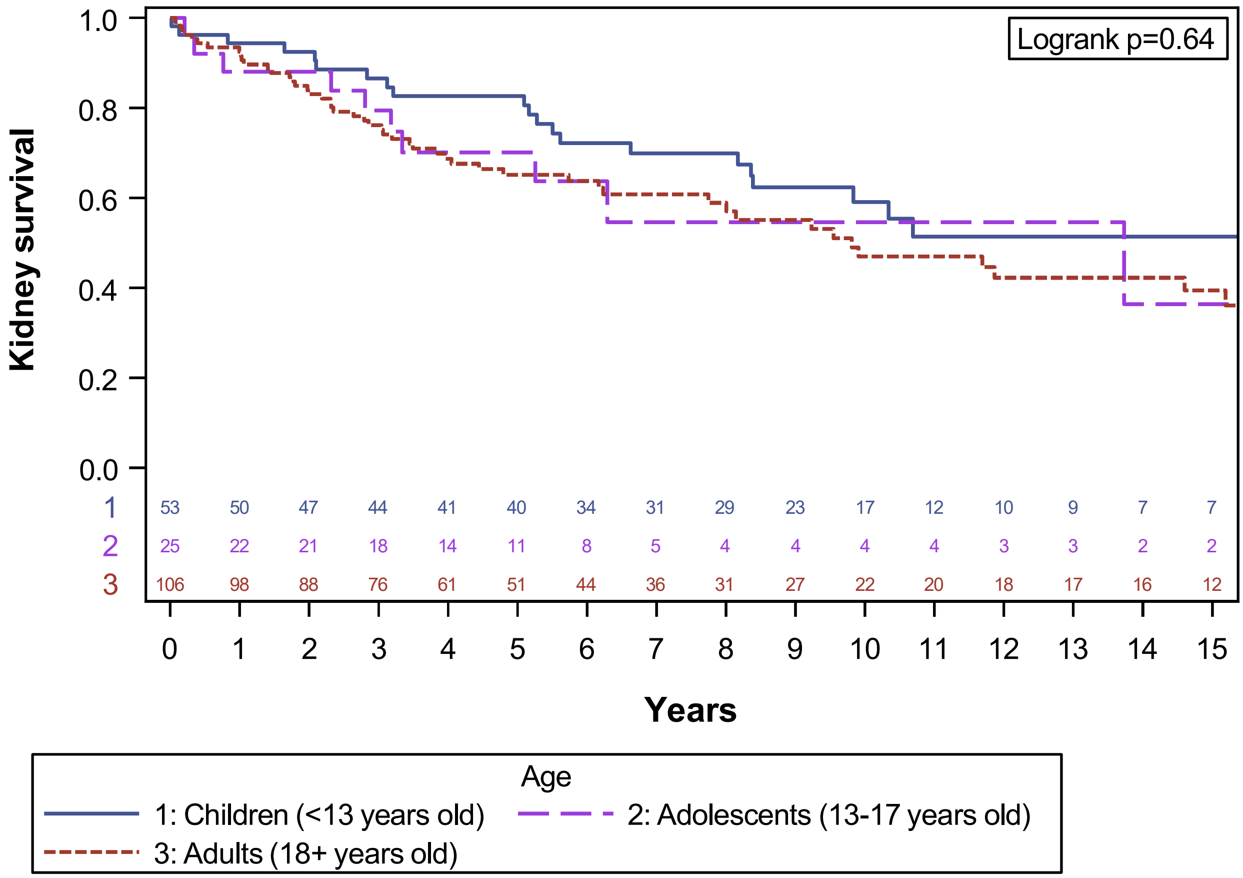

(n=184 participants; n=91 events)

© 2022 Gipson DS et al. *JAMA Network Open.*

eFigure 5. Time to ESKD or 40% Reduction in eGFR by Age: Significant Effect Modification by Sex in FSGS-CT

eFigure 5A. Time to ESKD or 40% reduction in eGFR by age: effect modification in Females - FSGS-CT Kaplan-Meier with logrank test

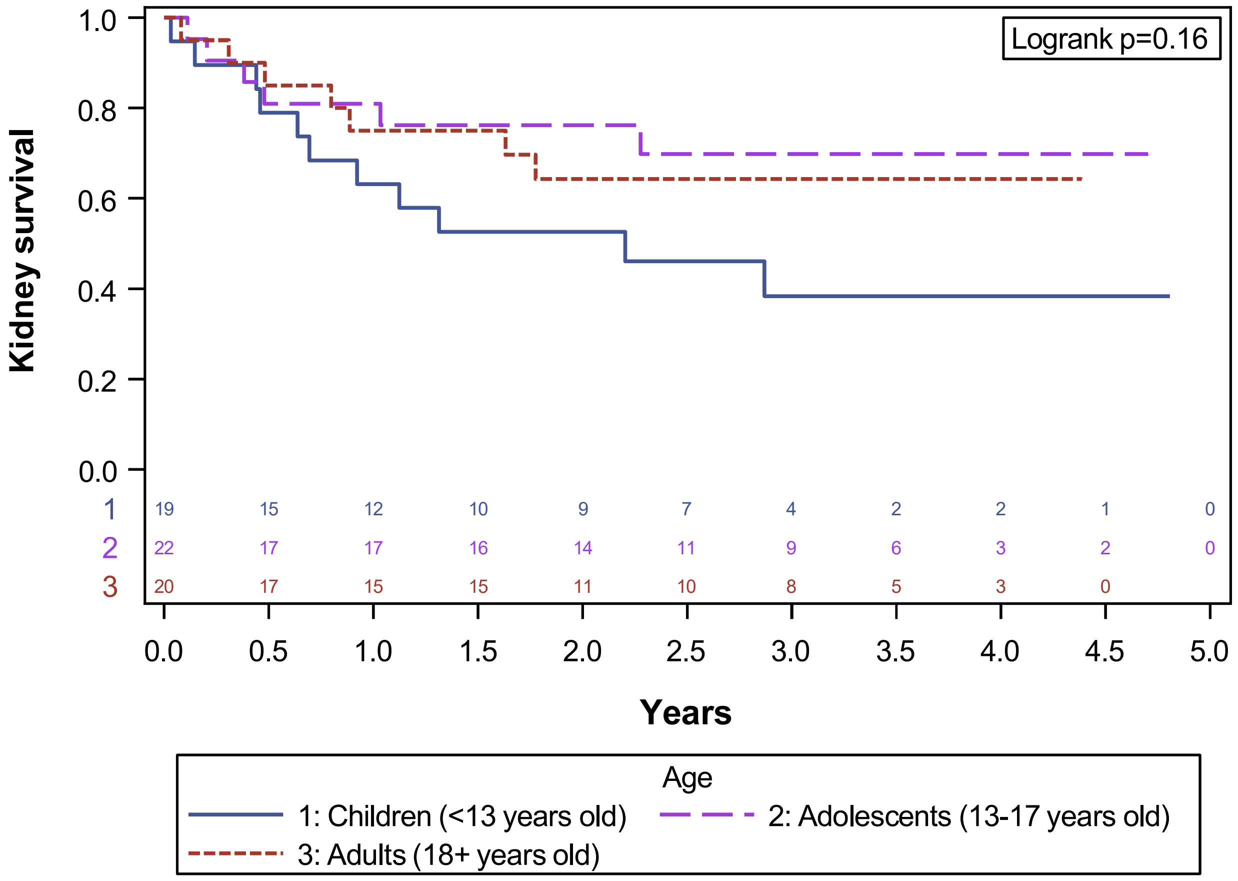

A: FSGS-CT females n=61 participants; n=24 events

© 2022 Gipson DS et al. *JAMA Network Open.*

eFigure 5B. Time to ESKD or 40% reduction in eGFR by age: effect modification in Males - FSGS-CT Kaplan-Meier with logrank test

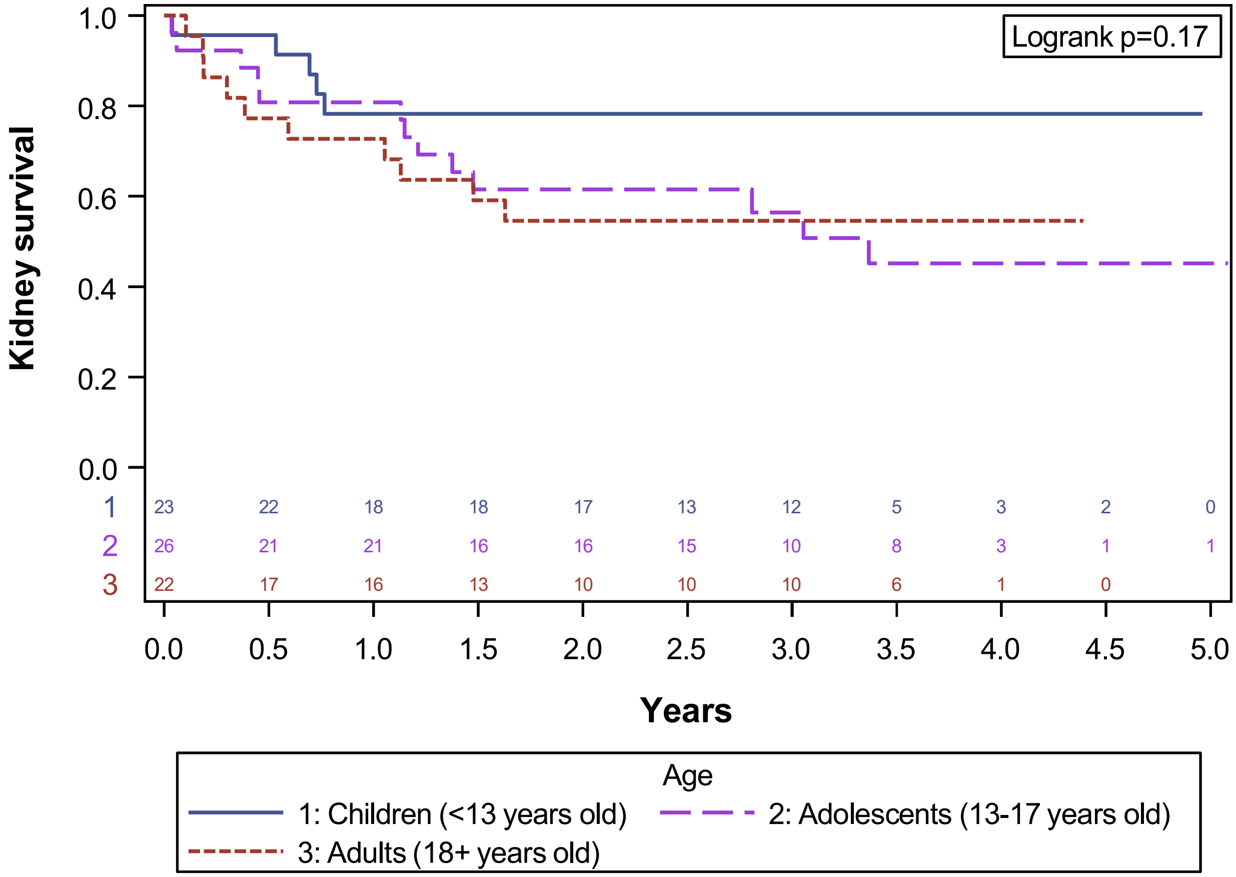

B: FSGS-CT males 71 participants; n=28 events

© 2022 Gipson DS et al. *JAMA Network Open.*

eFigure 6. Time to ESKD or 40% Reduction in eGFR by Age: Significant Effect Modification by Baseline UP:C Ratio in NEPTUNE

eFigure 6A. Time to ESKD or 40% reduction in eGFR by age: effect modification by baseline UP:C >3.5g/g – NEPTUNE Kaplan-Meier with logrank test

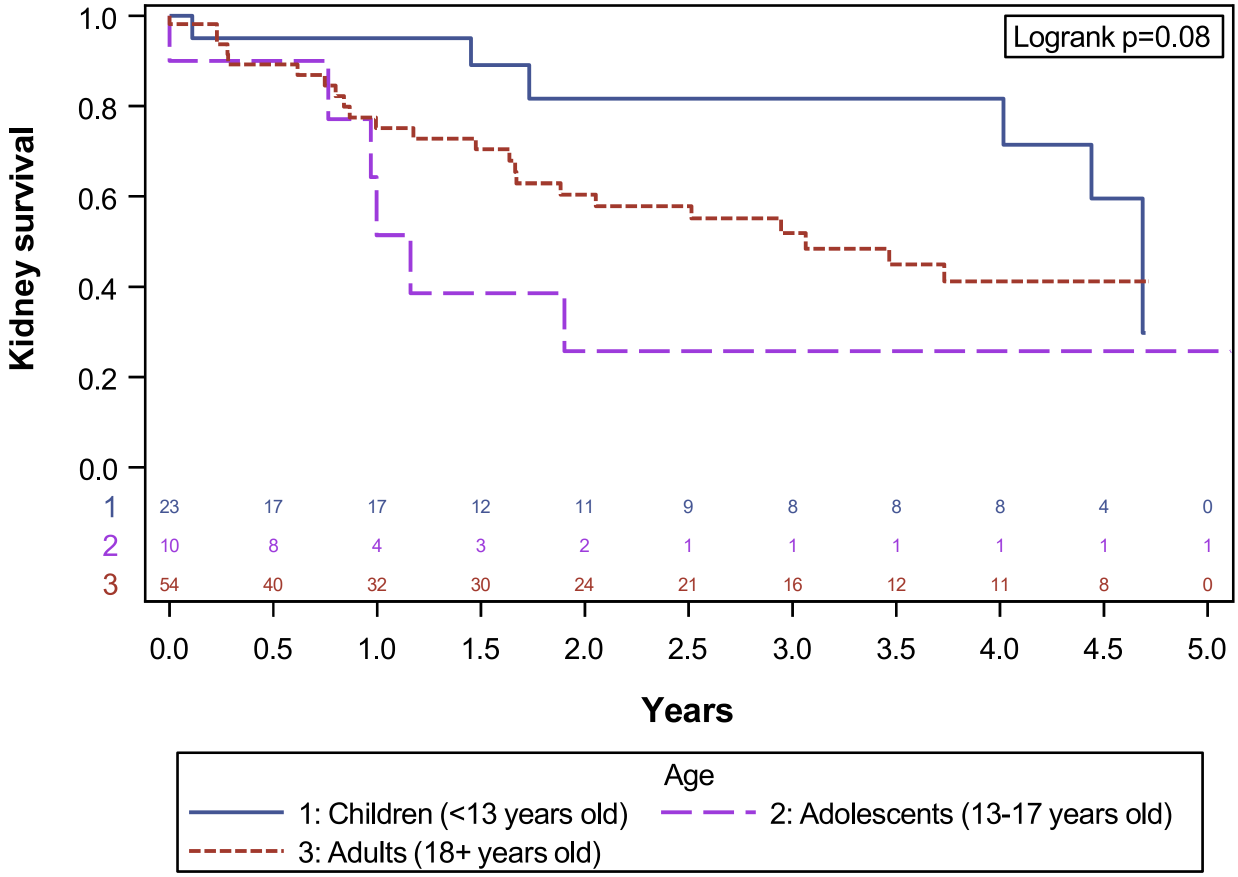

A: NEPTUNE UP:C>3.5g/g at baseline (n=87 participants; n=35 events)

© 2022 Gipson DS et al. *JAMA Network Open.*

eFigure 6B. Time to ESKD or 40% reduction in eGFR by age: effect modification by baseline UP:C ≤3.5g/g in NEPTUNE Kaplan-Meier with logrank test

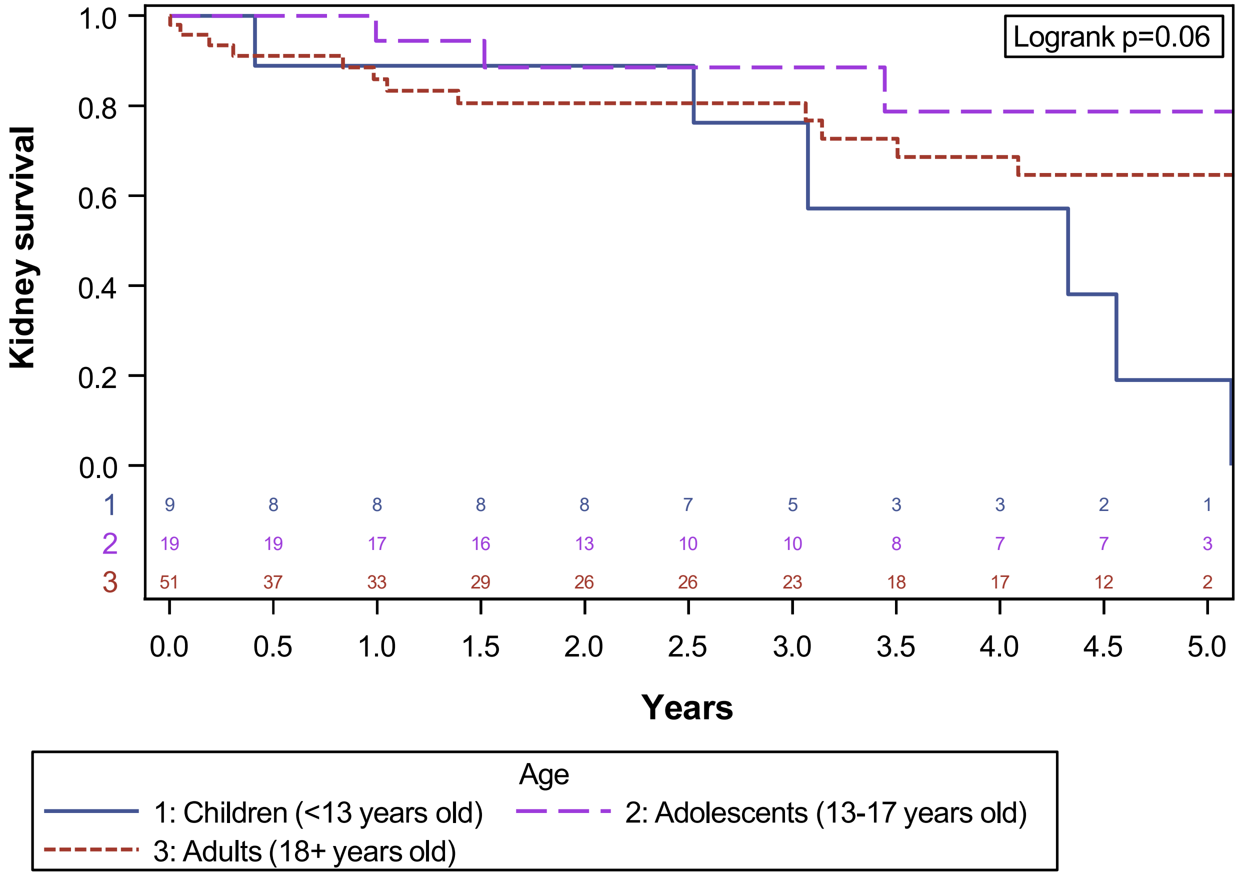

B: NEPTUNE UP:C≤3.5g/g at baseline (n=79 participants; n=21 events)

© 2022 Gipson DS et al. *JAMA Network Open.*

eFigure 7. Time To Complete or Partial Remission by Age (Partial Remission Defined as UP:C Ratio <3.5 g/g and 50% Reduction in UP:C Ratio From Baseline)

eFigure 75A. Time to complete/traditional partial remission by age – Neptune
Kaplan-Meier with logrank test

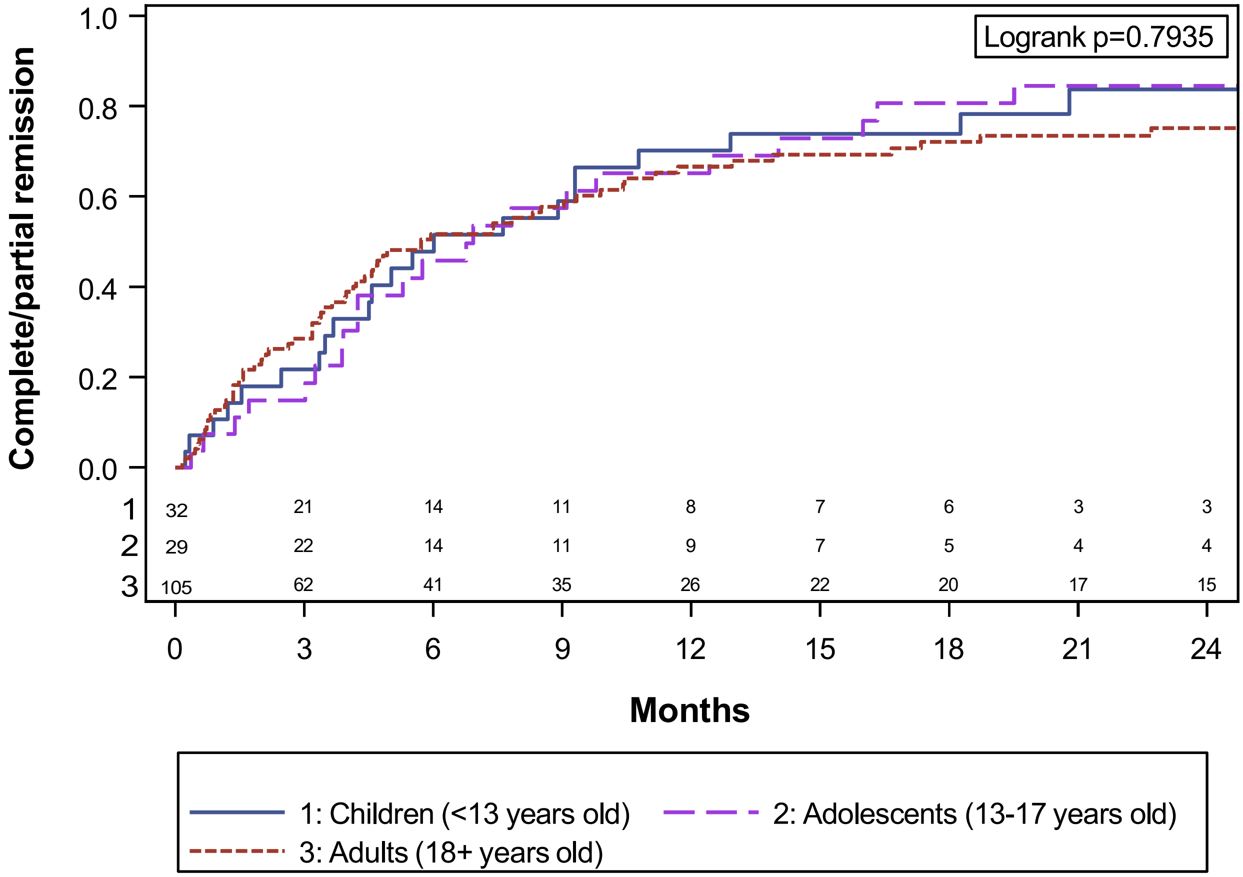

A: NEPTUNE n=166 participants; n=117 events
Traditional Partial remission=UP:C<3.5g/g and 50% reduction in UP:C from baseline

© 2022 Gipson DS et al. *JAMA Network Open.*

eFigure 7B. Time to complete/traditional partial remission by age – FSGS-CT
Kaplan-Meier with logrank test

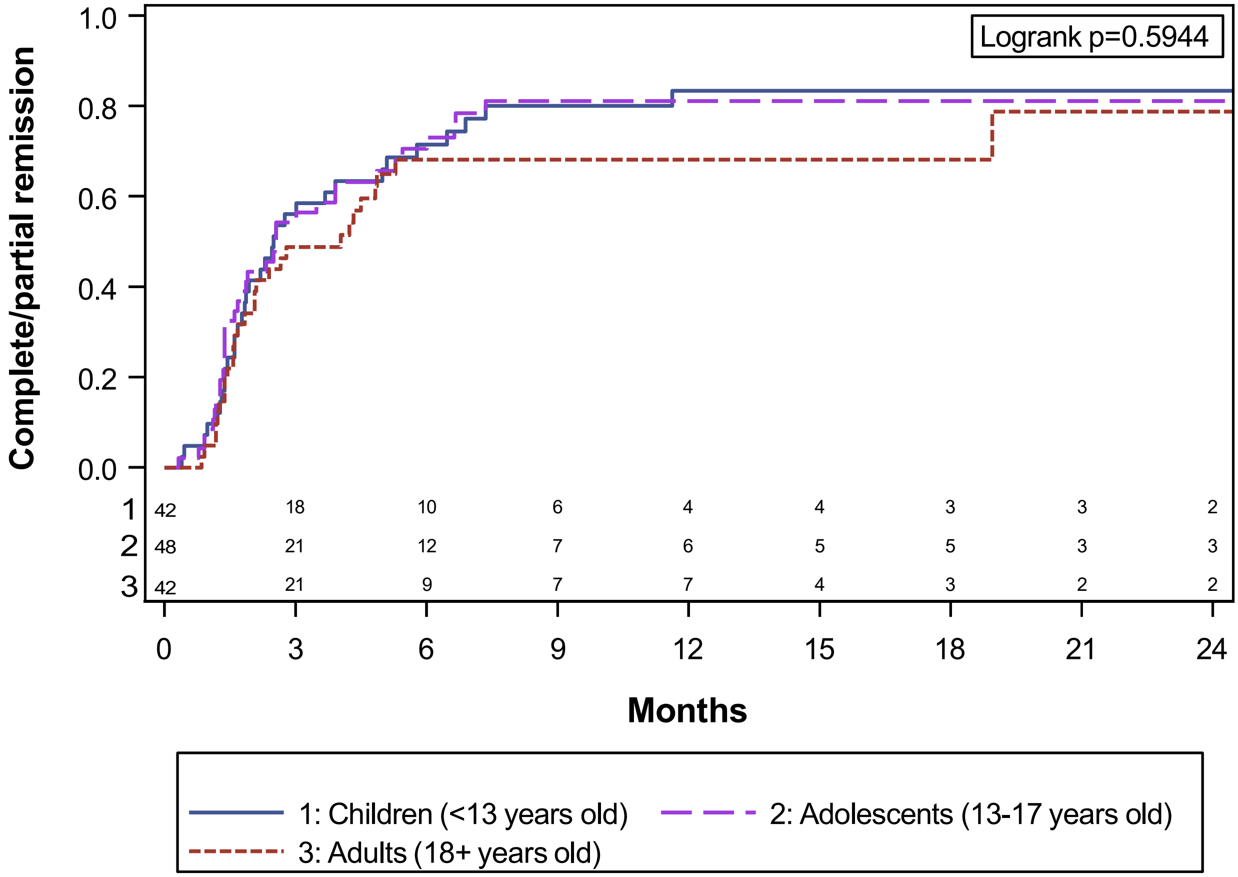

B: FSGS-CT n=132 participants; n=98 events
Traditional Partial remission=UP:C<3.5g/g and 50% reduction in UP:C from baseline

© 2022 Gipson DS et al. *JAMA Network Open.*

eFigure 7C. Time to complete/traditional partial remission by age – Pooled Kaplan-Meier with logrank test

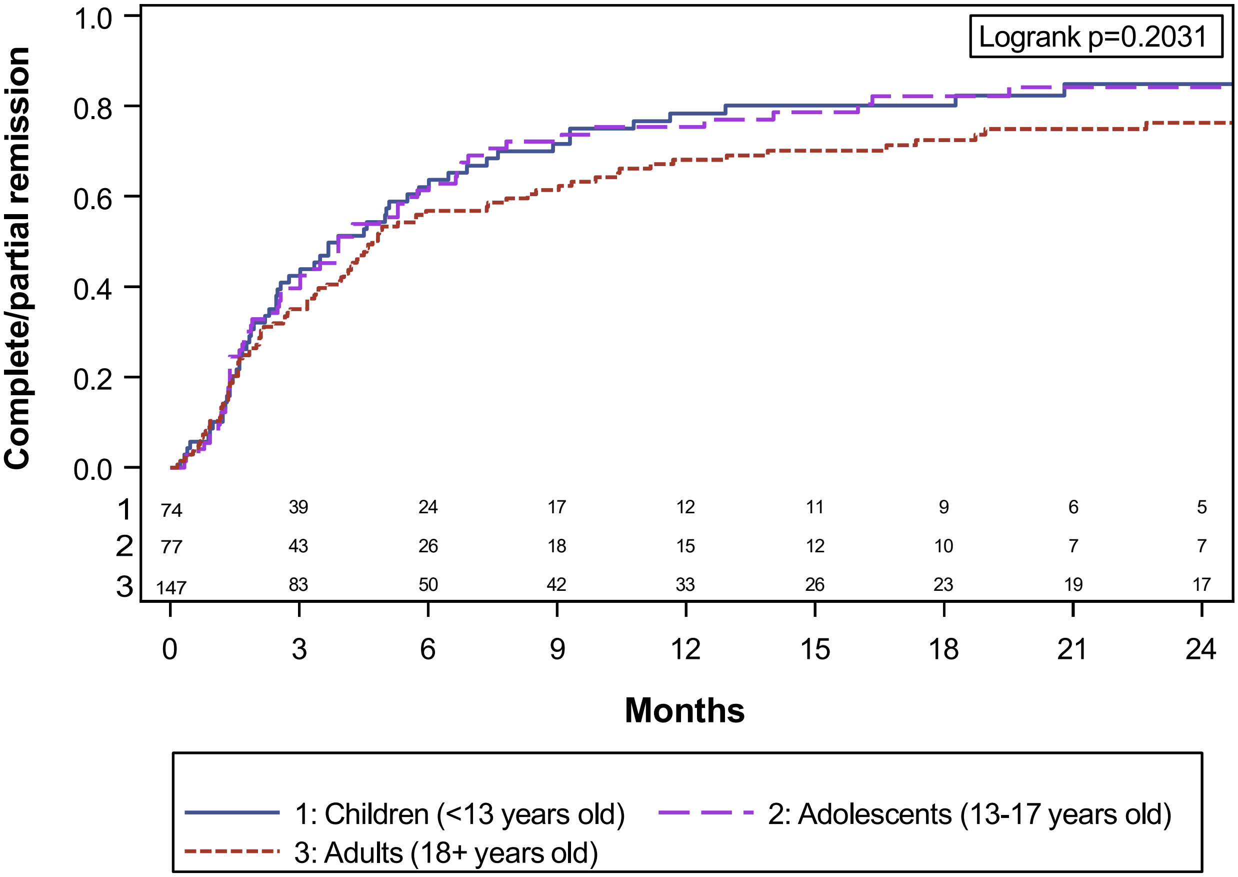

C: Pooled (NEPTUNE + FSGS-CT) n=298 participants; n=215 events
Traditional Partial remission=UP:C<3.5g/g and 50% reduction in UP:C from baseline

© 2022 Gipson DS et al. *JAMA Network Open.*
